# Oxidative Heck Reactions using Aryltrifluoroborates and Aryl *N*-Methyliminodiacetic Acid (MIDA) Boronates

**DOI:** 10.1002/open.201200007

**Published:** 2012-05-21

**Authors:** Jonas Sävmarker, Jonas Lindh, Peter Nilsson, Per J R Sjöberg, Mats Larhed

**Affiliations:** a Organic Pharmaceutical Chemistry, Department of Medicinal Chemistry, Biomedical Center (BMC), Uppsala UniversityBox 574, 751 23 Uppsala (Sweden) E-mail: Mats.larhed@orgfarm.uu.se; b Department of Physical and Analytical Chemistry, Biomedical Centre (BMC), Uppsala UniversityBox 599, 751 23 Uppsala (Sweden)

**Keywords:** Heck reactions, *N*-methyliminodiacetic acid (MIDA), oxidative reactions, palladium complexes, trifluoroborates

The oxidative version of the Mizoroki–Heck reaction was the first catalytic Heck reaction to be discovered. In 1968, Heck reported the palladium(II)-catalyzed arylation of olefins from phenylmercuric chloride, using catalytic amounts of copper(II) chloride assisted by oxygen for the regeneration of palladium(II).[1] Seven years later, Heck and Dieck reported the first palladium(II)-mediated vinylic substitution of organoboronic acid, using stoichiometric amounts of palladium acetate,2a today referred to as the oxidative Heck reaction.[2] This reaction is closely related to the classical Mizoroki–Heck reaction, but is initiated by a transmetallation step, rather than an oxidative addition process, and thereby requires an external reoxidizing agent in order to be catalytic. More recently, we and others have refined this reaction to become a base-free open-air process, and as a continuation, the use of aryltrifluoroborates (ArBF_3_K) as the aryl source were explored. Aryltrifluoroborates are considered to be competent alternatives to arylboronic acids in several palladium-catalyzed reactions such as the Suzuki–Miyaura[3] cross-coupling, the carbonylative Suzuki coupling,[4] and the hydroarylation[5] of α,β-unsaturated substrates.[6] Compared with boronic acids, the readily prepared[7] trifluoroborates benefit from longer shelf life and simplified purification. Recent advances in the preparation of organotrifluoroborates have improved the diversity and number of commercially available products, which further broaden the scope of the cross-coupling reactions.6e Hence, new methods to exploit the use of this class of protected boronic acids are of great interest.

Herein, we present a new base-free method for the palladium(II)-catalyzed coupling of aryltrifluoroborates and olefins. A microwave-assisted protocol quickly furnished the corresponding cinnamic acid esters with electron-deficient olefins at 120 °C using 1,3-bis(diphenylphosphino)propane (dppp) as the supporting bidentate ligand and *para*-benzoquinone (*p*-BQ) as the hydride acceptor.2k, [8] The electron-rich olefin, *n*-butyl vinyl ether, was regioselectively arylated to furnish branched products under the same conditions, with the addition of acetone as solvent and methanol as a co-solvent.

We have previously been able to react aryltrifluoroborates under similar conditions as the corresponding arylboronic acids by simply adding two equivalents of sodium trifluoroacetate (NaTFA).[9] Here, we aimed for an additive-free and pH-neutral protocol to activate these protected boronates. Electrospray ionization mass spectrometry (ESI–MS)[10] analysis was used to investigate the arylboron species formed in solution.[9, 11]

We initiated the study with the arylation of electron-deficient *n*-butyl acrylate, using potassium 4-methylphenyltrifluoroborate as the arylating agent and *p-*BQ as the hydride acceptor. With the olefin as the yield-determining reagent, a small microwave-assisted optimization regarding the choice of palladium(II) source, ligand and solvent was conducted. Complete consumption of the olefin was observed when employing 2 % Pd(TFA)_2_ and 3 % dppp in methanol at 120 °C (Scheme 1). The use of water, ethanol, *N*,*N*-dimethylformamide, acetone or acetonitrile resulted in incomplete conversion.

**Scheme 1:**
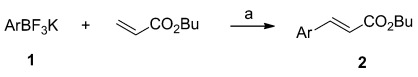
Oxidative Heck arylation of *n*-butyl acrylate. *Reagents and conditions*: a) Pd(TFA)_2_, dppp, *p*-BQ or O_2_, MeOH.

To investigate the scope of this reaction, a range of aryltrifluoroborates were tested (Table 1). Electron-rich **1 a** gave **2 a** as the sole product in 51 % yield after 20 min. However, doubling the reaction time provided complete conversion and an improved isolated yield of **2 a** (73 %; Entry 1, Table 1). The σ-donating functionalities in compounds **1 b** and **1 c** improved the outcome of the reaction and produced corresponding cinnamic esters **2 b** and **2 c** in 72 % and 80 % yield, respectively, after 20 min at 120 °C. Lowering the temperature to the solubility limit of substrate **1 c** (65 °C) furnished product **2 c** in 77 % yield after 18 h under *p*-BQ-free, open-air conditions (Entry 4, Table 1). Simple phenylation using **1 d** provided **2 d** in an excellent isolated yield (89 %). As expected, full chemoselectivity was obtained in using compounds **1 e** and **1 f** (Entries 7 and 8), where neither oxidation of the benzylic alcohol in **1 e** nor palladium(0)-mediated oxidative addition product of **1 f** were detected by GC-MS or LC-MS, and the desired products **2 e** and **2 f** were obtained in 71 % and 84 % yields, respectively. Reactions of electron-deficient compounds **1 g** and **1 h** were highly productive, furnishing **2 g** (87 %) and **2 h** (82 %). In addition, *para*-trifluoromethyl product **2 h** could be effectively produced under open-air conditions in 90 % isolated yield (Entry 11). *Ortho*-substituted **1 i** gave a surprisingly good isolated yield of **2 i** (91 %), indicating that the protocol has a good tolerance to steric hindrance. However, 2-naphthyltrifluoroborate gave a somewhat lower yield of **2 j** (70 %), mainly due to the competing proto-deboronation reaction. Heteroaromatic 2-furanyl suffered from incomplete conversion of *n*-butyl acrylate and produced a moderate 51 % yield of **2 k** at 120 °C (Entry 14). In all reactions, the expected terminally arylated *E*-alkene isomer was the major product, with the *Z*-isomer only observed in trace amounts.[11]

**Table 1:** Scope of the oxidative arylation of *n*-butyl acrylate[a]

Entry	ArBF_3_K	Product	*T* [°C]	Yield[b] [%]
1	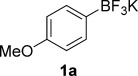	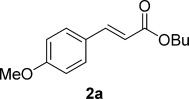	120	51
				73[c]
2	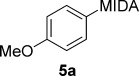	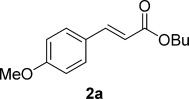	120	36[c]
3	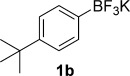	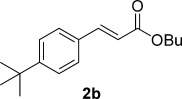	120	72
4	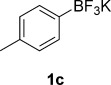	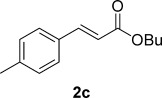	120 65	80
				77[d]
5	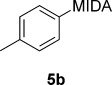	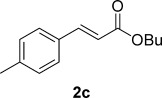	120	67[c]
6	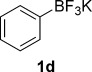	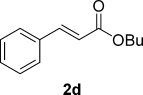	120	89
7	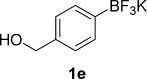	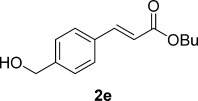	120	71
8	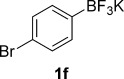	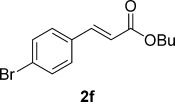	120	84
9	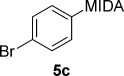	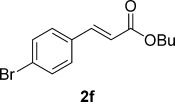	120	82[c]
10	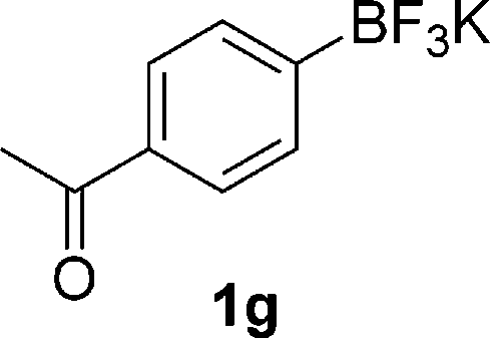	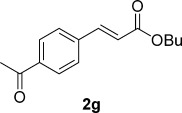	120	87
11	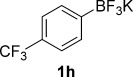	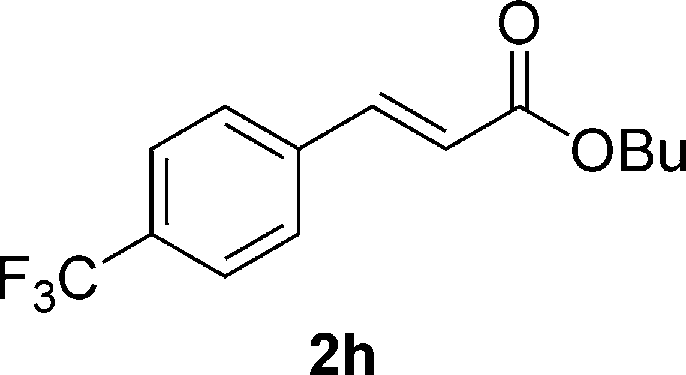	120 65	82
				90[d]
12	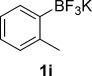	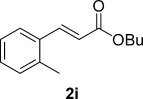	120	91
13	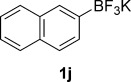	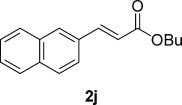	120	73
14	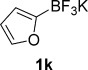	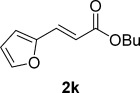	120	51

[a] *Reagents and conditions*: ArBF_3_K or ArMIDA (1.5 mmol), *n*-butyl acrylate (1.0 mmol), Pd(TFA)_2_ (0.02 mmol), dppp (0.03 mmol), *p*-BQ (1 mmol) and MeOH (3 mL), heated by microwave irradiation in a sealed vial at 120 °C for 20 min.

[b] Isolated yield; >95 % according to GC-MS and ^1^H NMR.

[c] Reaction was heated for 40 min.

[d] Reaction was carried out in MeOH (20 mL) and performed in an open vessel at 65 °C for 18 h.

Electron-rich alkoxy-substituted olefins, that is vinyl ethers, are of particular interest since regioselective arylation on the internal position affords,2e after hydrolysis, versatile acetophenone products (Scheme 2).[12, 13]

**Scheme 2:**
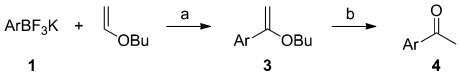
Regioselective oxidative Heck arylation of *n*-butyl vinyl ether. *Reagents and conditions*: a) Pd(TFA)_2_, dppp, *p*-BQ or O_2_, acetone/MeOH (2:1); b) 1 m aq HCl.

Accordingly, we decided to investigate the regioselectivity and productivity of arylations employing the same bidentate-ligand-promoted cationic protocol,[11] but with *n*-butyl vinyl ether as the olefin (Table 2). In general, slightly lower yields were obtained compared with *n*-butyl acrylate. Initially, the same protocol as for *n*-butyl acrylate was used, producing 36 % isolated yield of **3 c** in the microwave-assisted method and 43 % under conventional heating. The problem was identified as the decomposition of the vinyl ether as a result of the nucleophilic solvent. Among the solvents initially screened, acetone gave reasonable conversion and a 2:1 acetone/methanol mixture was found to be particularly productive.

**Table 2:** Scope of the oxidative arylation of *n*-butyl vinyl ether[a]

Entry	ArBF_3_K	Product	*T* [°C]	Yield[b] [%]
1	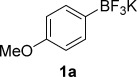	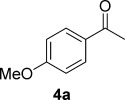	120	69
2	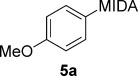	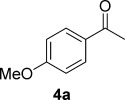	120	trace
3	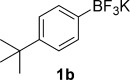	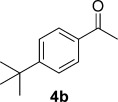	120	41
4	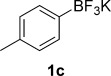	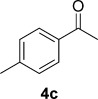	120	71
			120	36[c]
			65	43[d]
5	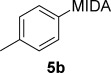	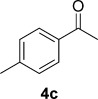	120	trace
6	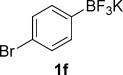	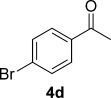	120	43
7	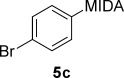	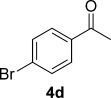	120	10
8	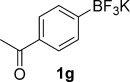	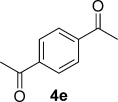	120	89
9	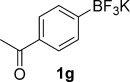	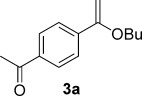	120	81[e]
10	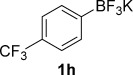	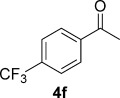	120	58

[a] *Reagents and conditions*: ArBF_3_K or ArMIDA (1.5 mmol), *n*-butyl vinyl ether (1.0 mmol), Pd(TFA)_2_ (0.02 mmol), dppp (0.03 mmol), *p*-BQ (1 mmol) and acetone/MeOH (2:1 mL), heated by microwave irradiation in a sealed vial at 120 °C for 20 min. Thereafter, 1 m aq HCl (2 mL) was added, and the mixture was stirred at RT for 1 h.

[b] Isolated yield; >95 % according to GC-MS and ^1^H NMR.

[c] MeOH (3 mL) alone was used as the solvent.

[d] Reaction was carried out in MeOH (20 mL) and performed in an open vessel at 65 °C for 18 h.

[e] Product isolated prior to hydrolysis.

With the modified protocol at hand, the scope of trifluoroborates **1** as arylating agents was investigated (Table 2). Reaction of electron-rich compound **1 a** gave, after hydrolysis, product **3 a** in 69 % yield after 20 min of microwave irradiation at 120 °C. *tert*-Butyl-substituted **1 b** furnished a moderate yield (41 %) of **4 b**, and no improvement was found by increasing the reaction time to 40 min. *para*-Tolyl compound **1 c** provided methyl ketone **4 c** in a 71 % isolated yield. Once again, the chemoselectivity was investigated by evaluating **1 f**, furnishing **4 d** in a moderate 43 % yield. In this case, we believe that the poor outcome might be a result of a slow insertion rate rather than interfering palladium(0) oxidative addition of the aryl bromide, since the identified by-products (4,4′-dibromobiphenyl and protodeboronated phenyl bromide) both have the halogen functionality intact. Electron-deficient **1 g** provided **4 e** in an excellent yield (89 %), and nonhydrolyzed product **3 a** was isolated in a similar yield (81 %; Entry 9, Table 2). The σ-withdrawing trifluoromethyl substituent on **1 h** decreased the yield to a disappointing 58 %, similar to previous observations.2e, [14] In all reactions the expected regioselectivity was observed, providing the internal product exclusively before hydrolysis.[15]

Recently, a new type of *N*-methyliminodiacetic acid (MIDA)-protected organic boronic acids has been introduced, named MIDA boronates.[16] These boronic acid surrogates (**5**) are readily deprotected at room temperature under mild hydrolytic conditions, and we decided to investigate how these novel alternatives compare with the corresponding trifluoroborates (**1**) in our protocol using *n*-butyl acrylate and *n*-butyl vinyl ether (Scheme 3).

**Scheme 3:**
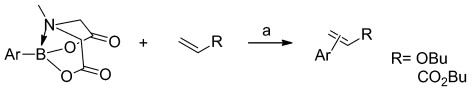
Oxidative Heck arylation using MIDA boronate esters. *Reagents and conditions*: a) Pd(TFA)_2_, dppp, *p*-BQ, MeOH or acetone/MeOH (2:1).

Reactions with electron-deficient *n*-butyl acrylate (Table 1) showed that the MIDA substrates furnish useful but somewhat lower yields than the corresponding trifluoroborates (c.f., Entries 1–2, 4–5 and 8–9). Similar chemoselectivities with aryl-bromine-containing substrates **1 f** and **5 c** were also obtained (c.f., Entries 8 and 9). Surprisingly, employing the electron-rich olefin *n*-butyl vinyl ether as a coupling partner to the aryl-MIDA substrates provided **4** in a yield of only 0–10 % (Entries 2, 5 and 9, Table 2). One possible explanation could be a slower hydrolysis to the corresponding active boron complex under these less basic conditions allowing a substantial competing decomposition of the sensitive olefin.

There are many reported studies focused on the nature of the palladium(II) active boronate species of aryl boronic acids,[17] and for quite some time, aryltrihydroxyborates have been considered to be the active boronate to undergo transmetallation in coupling reactions.[18] These tentative intermediates, formed under basic conditions, are stable compounds and are now made commercially available. Recently, Hartwig et al. published a study of the two possibly competing pathways for transmetallation with the intact boronic acid as the favorable substrate.[19] Investigations regarding the mechanism behind the activation of aryltrifluoroborates have also been reported.[20] Recently, it was reported that the lowest energetic route for transmetallation under common cross-coupling conditions was the complete hydrolysis to the corresponding boronic acid.[21] The reported studies were all conducted under palladium(0)-catalyzed conditions where the aryl–palladium(II) complex is generated by an initial oxidative addition, which subsequently undergoes transmetallation, and not an inorganic palladium(II) catalyst, as assumed in the oxidative Heck mechanism. In relation to our present protocol, the relative energy barrier for transmetallation could be similar and therefore the reactivity analogous, but it must be stressed that our conditions do not contain any added base nor any added water, except for trace amounts in the solvents and on the surface of the glassware used. These observations encouraged us to investigate the formation of different boron species in an ongoing reaction.

We have previously been successful in identifying cationic palladium complexes by online ESI–MS analysis,[9], 11d, f, [22] and we sought to use the same methodology to detect the various boron species. In order to detect the arylboronates formed, the reaction providing **2 c** (Entry 4, Table 1) was set up at room temperature. This reaction was found to require around five days to reach complete conversion. A small amount of reaction mixture was withdrawn after 12 h, diluted with methanol and analyzed using ESI–MS in the negative mode[23] (Figure 1). A number of negatively charged arylboron species were identified, among them, a weak signal from the deprotonated boronic acid.

**Figure 1:**
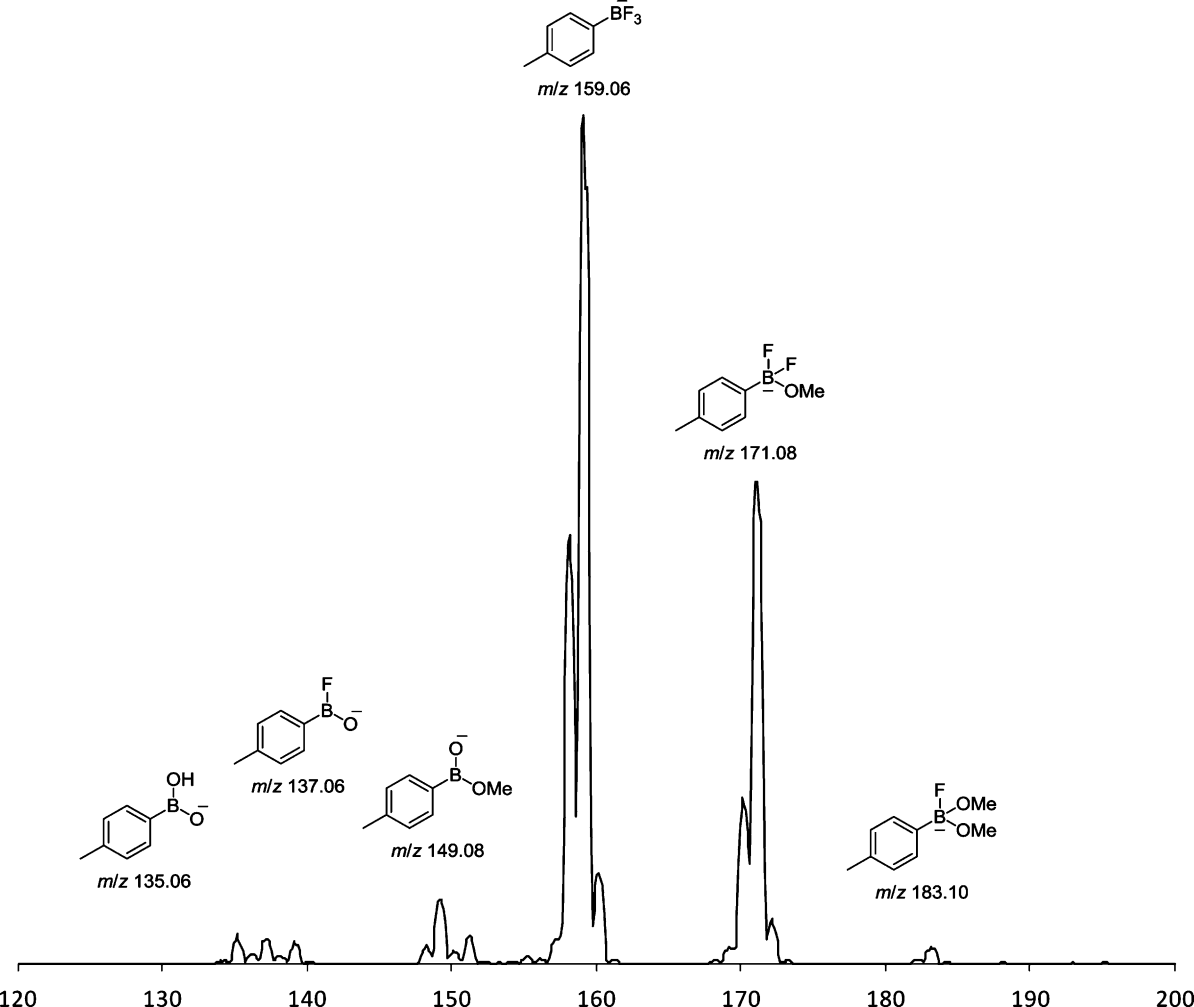
ESI–MS scan of an ongoing reaction (60 °C, 30 min) and the proposed cationic boron species.

In order to detect any neutral intermediates, permanently charged potassium 4-trimethylammonium phenyltrifluoroborate iodide was employed.[24] Due to poor solubility of this substrate in methanol, the reaction was heated to 60 °C and a small amount of reaction mixture was withdrawn after just 30 min to be diluted and analyzed. As depicted in Figure 2, several positively charged organoboron species were detected using ESI–MS, among them, the important ArBF_2_ intermediate in the dissociative hydrolysis of the trifluoroborates.20b

**Figure 2:**
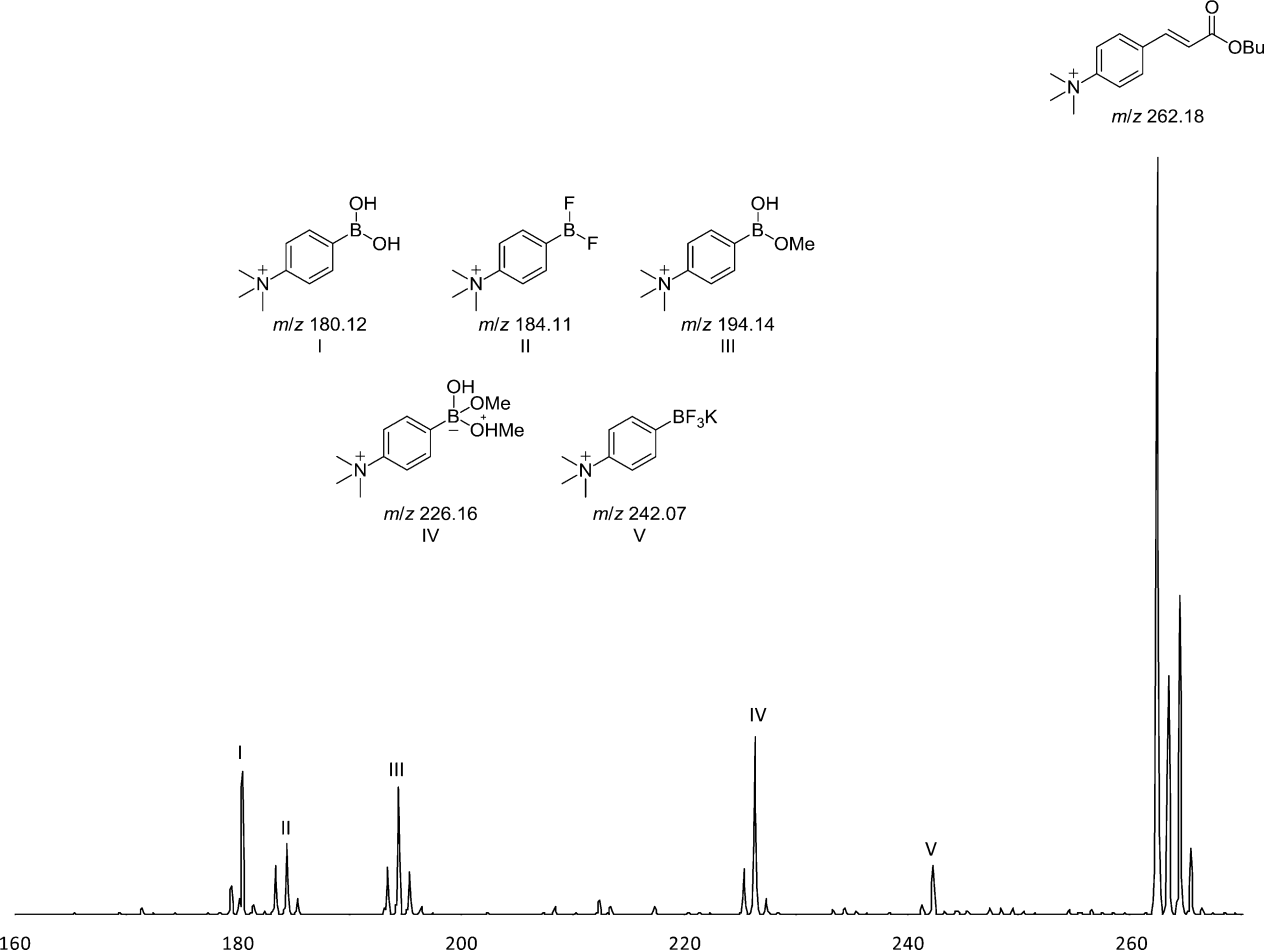
ESI–MS scan of an ongoing reaction (RT, 12 h) and the proposed anionic boron species.

Furthermore, combining the information from cationic and anionic ESI–MS data, it became evident that the boronic acid is present in the reaction cocktail. This observed hydrolysis to the boronic acid is in agreement with previously published findings that even without adding base, hydrolysis is observed when using a glass-walled reaction vessel.[21, 25] Hence, it would be quite reasonable to speculate that it is the formed arylboronic acid that most easily participates in the transmetallation step, even under our pH-neutral and water-deficient conditions.

A proposed general mechanism is depicted in Scheme 4. We assume that the palladium retains its oxidation state throughout the whole cycle and that *p*-BQ act as a hydride acceptor. In the cases where *p*-BQ-free conditions are used under air, we believe it is dioxygen that operates as the hydride acceptor forming hydrogen peroxide.[7] The assumption that palladium(0) is never generated in the catalytic cycle is supported by the fact that aryl bromides are not activated under our conditions (Entries 8 and 9, Table 1; Entry 6, Table 2). Furthermore, the high relative intensities of the palladium(II)–hydride species indicate the non-immediate reductive elimination to free palladium(0) (see the Supporting Information).

**Scheme 4:**
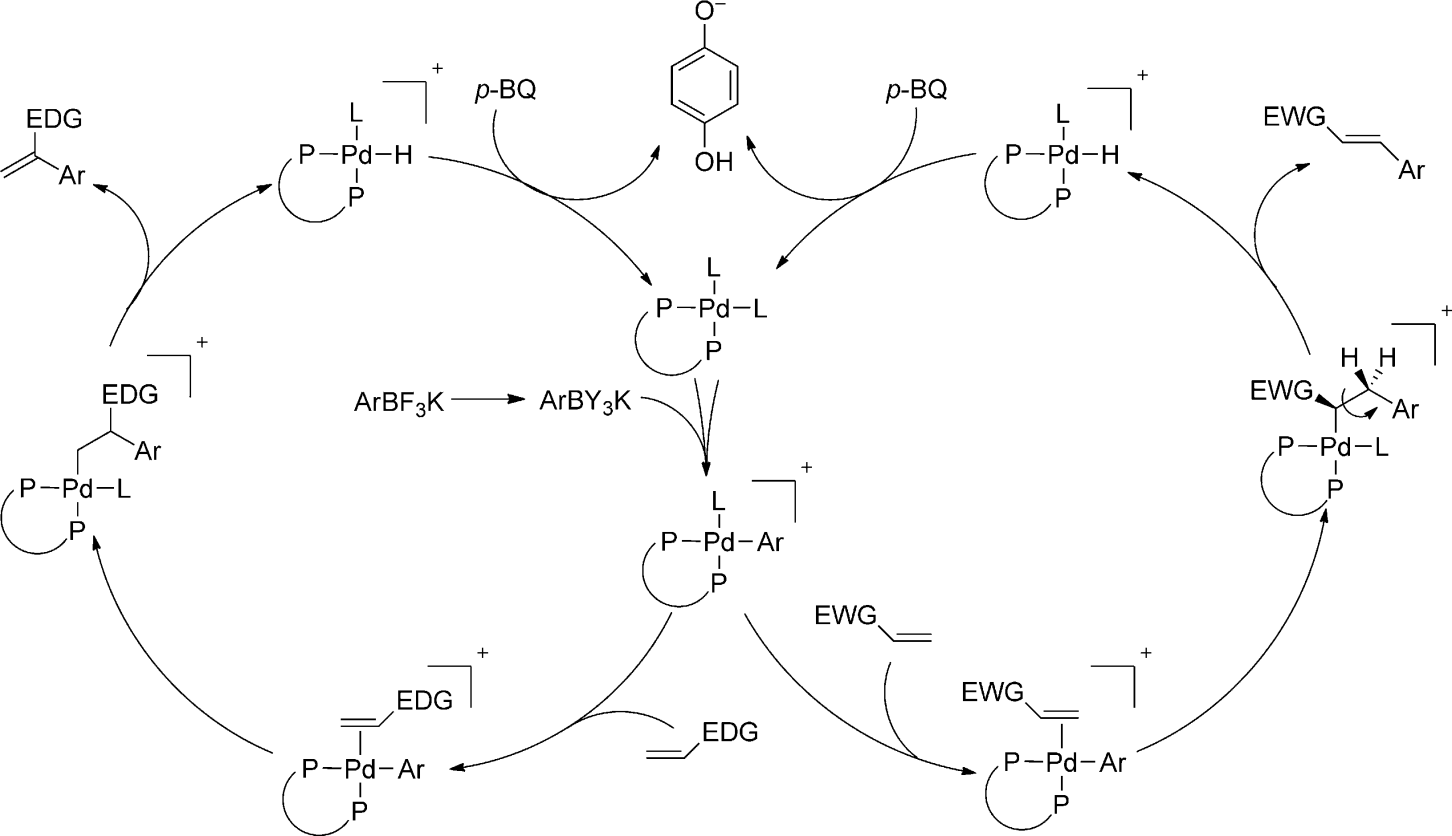
Proposed mechanism for the palladium(II)-catalyzed oxidative Heck reaction. Abbreviations: electron-withdrawing group (EWG); electron-donating group (EDG); Pd indicates Pd^II^; Y is F, OH or OMe; L indicates ligand or solvent (trifluoroacetic acid (TFA) or 4-hydroxyphenolate), 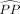 indicates dppp.

In conclusion, we have developed a general and robust oxidative Heck methodology for selective terminal arylation of *n*-butyl acrylate and internal arylation of *n*-butyl vinyl ether employing aryltrifluoroborates and aryl-MIDA boronates. Furthermore, the arylboronic acid was detected in the reaction cocktail by ESI–MS and is suggested to be the species that undergoes the transmetallation step. The oxidizing agent *p*-BQ or O_2_ is likely to act as a hydride acceptor, thus preserving the palladium(II) oxidation state throughout the catalytic cycle.

## Experimental Section

**General procedure for the reaction with** ***n*****-butyl acrylate**: A 5 mL microwave transparent vial or a 50 mL round bottomed flask was charged with Pd(TFA)_2_ (6.6 mg, 0.02 mmol), dppp (12.4 mg, 0.03 mmol) and MeOH (3 or 20 mL), and the solution was treated with ArBF_3_K or Ar–MIDA boronate (1.5 mmol), *n*-butyl acrylate (128 mg, 1.0 mmol) and *p*-BQ (108 mg, 1.0 mmol). The vial was equipped with a stirrer, capped and exposed to microwave heating for 20–40 min at 120 °C or left open, without the addition of *p*-BQ, for conventional heating during 18 h at 65 °C. The reaction container was allowed to reach RT (if needed, the volume was decreased by concentrating the solvent down to about 3 mL), and the mixture was extracted (×3; CH_2_Cl_2_/0.1 m aq NaOH, 20:20 mL). The combined organic layers were concentrated in vacuo, and the crude product was purified by silica column chromatography (*iso*-hexane/EtOAc, 9:1) to provide the desired product.

**General procedure for the reaction with** ***n*****-butyl vinyl ether**: A 5 mL microwave transparent vial was charged with Pd(TFA)_2_ (6.6 mg, 0.02 mmol), dppp (12.4 mg, 0.03 mmol) and acetone/MeOH (2:1; 3 mL), and the solution was treated with aryltrifluoroborate or aryl–MIDA boronate (1.5 mmol) and *n*-butyl vinyl ether (100 mg, 1.0 mmol) and *p*-BQ (108 mg, 1.0 mmol). The vial was equipped with a stirrer, capped and exposed to microwave heating for 20 min at 120 °C. The reaction mixture was then allowed to reach RT, and 1 m aq HCl (2 mL) was added. The mixture was stirred for 1–2 h at RT, and then extracted (×3; CH_2_Cl_2_/0.1 m aq NaOH, 30:30 mL). The organic layers were combined and concentrated in vacuo, and the crude product was purified by silica column chromatography (*iso*-hexane/EtOAc, 9:1) to provide the desired product.

**Supporting Information**: Full experimental details, characterization data for compound **2 e**, and NMR and MS spectra are available in the Supporting Information.

## Supplementary material

Detailed facts of importance to specialist readers are published as ”Supporting Information”. Such documents are peer-reviewed, but not copy-edited or typeset. They are made available as submitted by the authors.
