# Supplementary material for: Oxidative Heck Reactions using Aryltrifluoroborates and Aryl N-Methyliminodiacetic Acid (MIDA) Boronates
Source: ChemistryOpen. 2012 May 21;1(3):140–6. doi: 10.1002/open.201200007 (PMC3922452; doi:10.1002/open.201200007)

Supporting Information

© Copyright Wiley-VCH Verlag GmbH & Co. KGaA, 69451 Weinheim, 2012

Oxidative Heck Reactions using Aryltrifluoroborates and Aryl *N*-Methyliminodiacetic Acid (MIDA) Boronates

Jonas Sävmarker,[a] Jonas Lindh,[a] Peter Nilsson,[a] Per J. R. Sjöberg,[b] and Mats Larhed*[a]

open_201200007_sm_miscellaneous_information.pdf

EXPERIMENTAL SECTION S-2

General Information and Materials S-2

Electrospray Mass Spectrometry (ESI-MS) Experiments S-2

General procedure for oxidative Heck reactions using *n*-butyl acrylate S-2

General procedure for oxidative Heck reactions using *n*-butyl acrylate in an open flask S-3

General procedure for oxidative Heck reactions using *n*-butyl vinyl ether S-3

Characterization Data for Compounds 2e S-4

(*E*)-butyl 3-(4-*tert*-butylphenyl)acrylate (2e) S-4

References S-7

^{1}H NMR, ^{13}C NMR and GC-MS spectra of compounds 2a-d, f-k S-8

GC-MS spectra of compounds 3a and 4a-f S-28

ESI-MS-(+) spectra S-35

EXPERIMENTAL SECTION

General Information and Materials

The microwave heating was performed in a *Biotage* Initiator single mode reactor, which produces controlled irradiation at 2450 MHz. The reaction temperature was determined using the built-in online IR sensor. Microwave mediated reactions were performed in sealed *Smith* process vials designed for 2-5 mL reaction volumes. Analytical TLC was performed using *Merck* aluminium-backed 0.2 mm silica gel 60 F-254 plates and Visualization was done with UV light (λ = 254 nM). Silica gel 60 was purchased from *Merck*. NMR spectra were recorded on a *Varian* Mercury plus at 25 °C and 400 MHz for ^{1}H and 101 MHz for ^{13}C. Chemical shifts (δ) are reported in ppm and referenced indirectly to TMS via the solvent (or residual solvent) signals. Low-resolution mass spectra were recorded on a GC-MS instrument equipped with a CP-Sil 8 CB capillary column (30 m x 0.25 mm, 0.25 μm) operating at an ionization energy of 70 eV. The oven temperature (GC) was 70-300 °C. All starting materials, reagents and solvents are commercially available and were used as received. Products **2a**,[1] **2b**,[2] **2f**,[1] **2g**,[3] **2i**,[4] **2j**,[5] **2k**[6] and **3a**[7] have been previously reported and characterized. Styrenyl product **2e** is to the best of our knowledge a new compound. Products **2c-d**, **2h** and **4a-f** are commercially available.

Electrospray Mass Spectrometry (ESI-MS) Experiments

An API III+ triple-quadrupole mass spectrometer equipped with an articulated IonSpray interface was used in this study. The reaction mixture was typically diluted 10 times with methanol after 12 hours at room temperature or 30 min at 40-60 °C, stirring in an open vessel and introduced by continuous infusion with the aid of a syringe pump at a flow-rate of 5 µL/min through a fused silica capillary (50 µm inner diameter and 184 µm outer diameter). The fused silica capillary was centered in a stainless steel capillary counter assembly, which also served as the ESI high voltage contact. The nebulizer gas flow was set to 0.5 L/min. The flow rate of dry nitrogen counter-current curtain gas (heated to 60 °C) was 1.2 L/min over the sampling orifice. The mass spectrometric parameters were as follows: ion spray voltage (ISV) 3500 V, interface plate voltage (IN) 650 V, orifice lens voltage (OR) 50 V, and AC entrance rod (R0) 30 V. Mass spectral data was typically recorded by scanning the 100-800 u region with a dwell time of 1-2 ms and a step size of 0.1 u in multi channel acquisition mode (MCA summation of 10-20 scans). Mass scale calibration was performed using a polypropylene glycol solution (PPG). During the MS-MS experiments the collision energy was set to 20 eV. The collision gas was argon with 99.9999% purity. The collision gas thickness was $2{\cdot}10^{15}$ molecules$\cdot$cm^{-2}.

General procedure for oxidative Heck reactions using *n*-butyl acrylate

A 5 mL microwave transparent vial was charged with $Pd(TFA)_2$ (6.6 mg, 0.02 mmol), dppp (12.4 mg, 0.03 mmol) and 3 mL MeOH and stirred for 10 min. Then, *n*-butyl acrylate (128 mg, 1.0 mmol), aryltrifluoroborate **1** or aryl-MIDA boronate **5** (1.5 mmol) and *p*-BQ (108 mg, 1.0 mmol) were added to the vial which was thereafter capped and exposed to microwave heating for 20-40 min at 120 °C. The reaction mixture was

thereafter cooled to room temperature and extracted three times (dichloromethane:0.1 M NaOH (aq.)). The organic layers were combined and concentrated. The crude product was thereafter purified by column chromatography (silica gel) using iso-hexane:EtOAc (9:1) as eluent to give the isolated products. Temperature and pressure profiles for the reaction presented in Table 1, entry 4 is presented below.

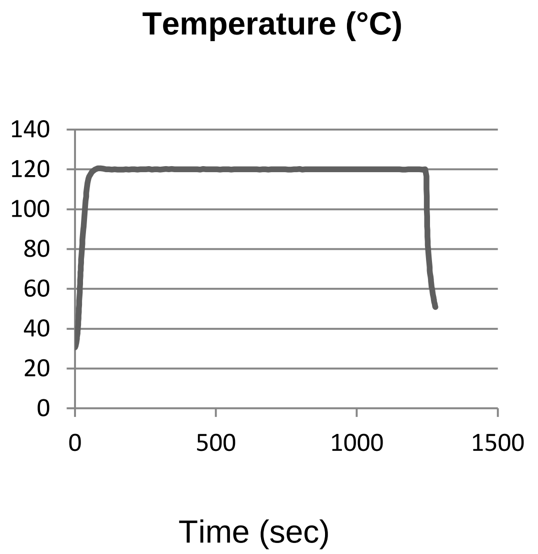

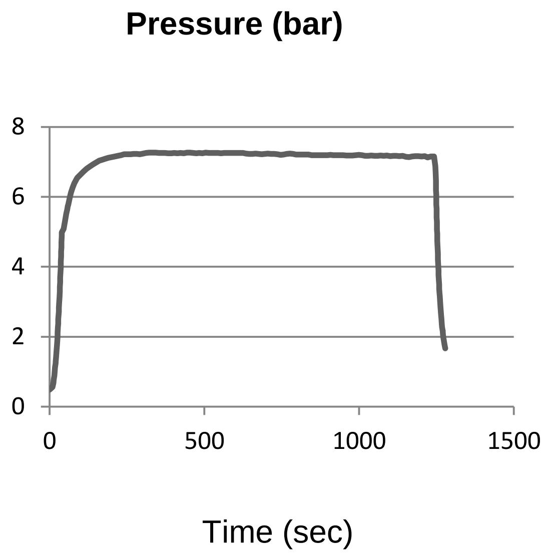

General procedure for oxidative Heck reactions using *n*-butyl acrylate in an open flask

A 50 mL round bottomed flask was equipped with an air condenser (150 mm) and charged with $Pd(TFA)_2$ (6.6 mg, 0.02 mmol), dppp (12.4 mg, 0.03 mmol), *n*-butyl acrylate (128 mg, 1.0 mmol), $ArBF_3K$ (**1**) (1.5 mmol) and 20 mL MeOH. Then the mixture was stirred in an oil-bath at 65 °C for 18 h. The reaction mixture was thereafter concentrated to approx. 3 mL and extracted three times (dichloromethane:0.1 M NaOH (aq)). The organic layers were combined and concentrated. The crude product was thereafter purified by column chromatography (silica gel) using iso-hexane:EtOAc (9:1) as eluent to give the isolated products.

General procedure for oxidative Heck reactions using *n*-butyl vinyl ether

A 5 mL microwave transparent vial was charged with $Pd(TFA)_2$ (6.6 mg, 0.02 mmol), dppp (12.4 mg, 0.03 mmol) and 3 mL acetone:MeOH (2:1) and stirred for 10 min. Then, *n*-butyl vinyl ether (100 mg, 1.0 mmol), aryltrifluoroborate **1** or aryl-MIDA boronate **5** (1.5 mmol) and *p*-BQ (108 mg, 1.0 mmol) were added to the vial which was thereafter capped and exposed to microwave heating for 20 min at 120 °C. The reaction mixture was then allowed to reach room temperature and 2 mL HCl (1M, aq.) was added. The mixture was stirred for 1-2 h at rt followed by three extractions (dichloromethane:NaOH (0.1M, aq) 30:30 mL). The organic layers were combined and concentrated. The crude product was thereafter purified by silica column chromatography (*iso*-hexane/EtOAc 9:1) to provide the desired product.

Characterization Data for Compounds 2e

(*E*)-butyl 3-(4-*tert*-butylphenyl)acrylate (2e)

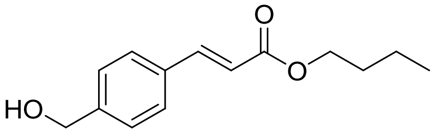

Following the general procedure for oxidative Heck reactions using n-butyl acrylate provided the product as a colorless oil in the yield stated in table 1. ^{1}H NMR (400 MHz, $CDCl_3$): δ 0.96 (t, ^3J = 7.4 Hz, 3H), 1.38-1.48 (m, 2 H), 1.64-1.72 (m, 2H), 2.20 (br s, 1H), 4.19 (t, ^3J = 6.6 Hz, 2H), 4.69 (s, 2 H), 6.40 (dm, ^3J= 16.0 Hz, 1H), 7.36 (m, 2H), 7.49 (m, 2H), 7.64 (dm, ^3J= 16.0 Hz, 1H); ^{13}C NMR (101 MHz, $CDCl_3$): δ 14.0, 19.4, 31.0, 64.7, 65.0, 118.3, 127.5, 128.5, 133.9, 143.5, 144.5, 167.4; MS (70 eV): *m/z* (%): 235 (37) [M^++H], 234 (16) [M^+], 216 (25), 178 (62), 61 (29), 160 (19), 131 (100), 103 (46), 77 (34). HRMS (ESI): *m/z* calcd for $C_{14}H_{19}O_3$+: 235,1329 [M^++H]; found: 235,1309.

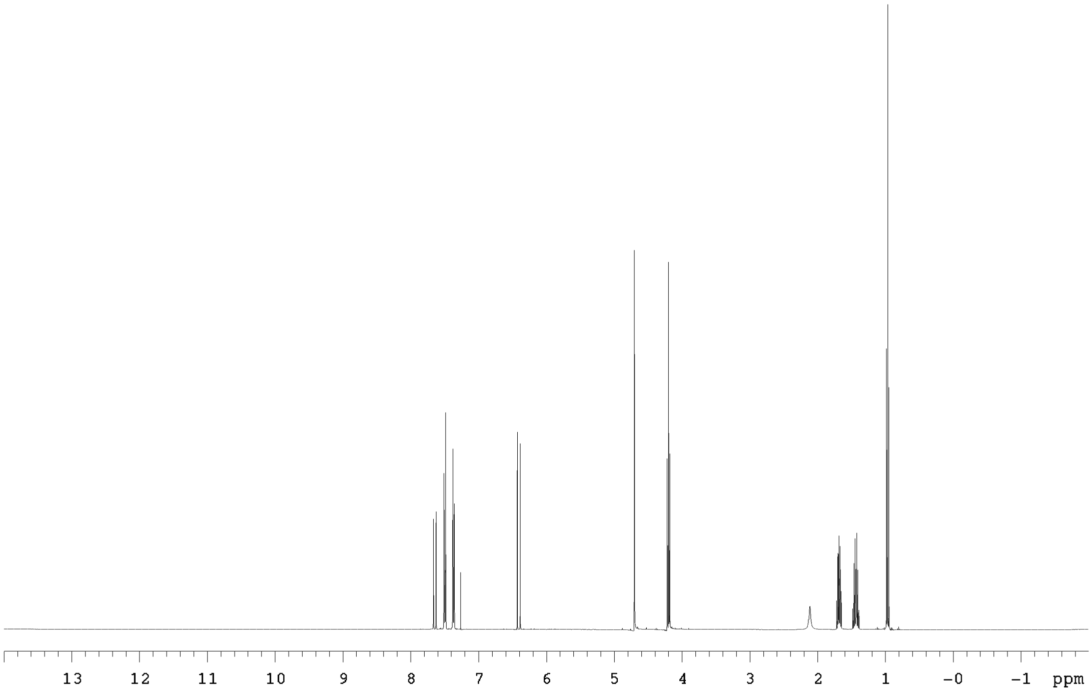

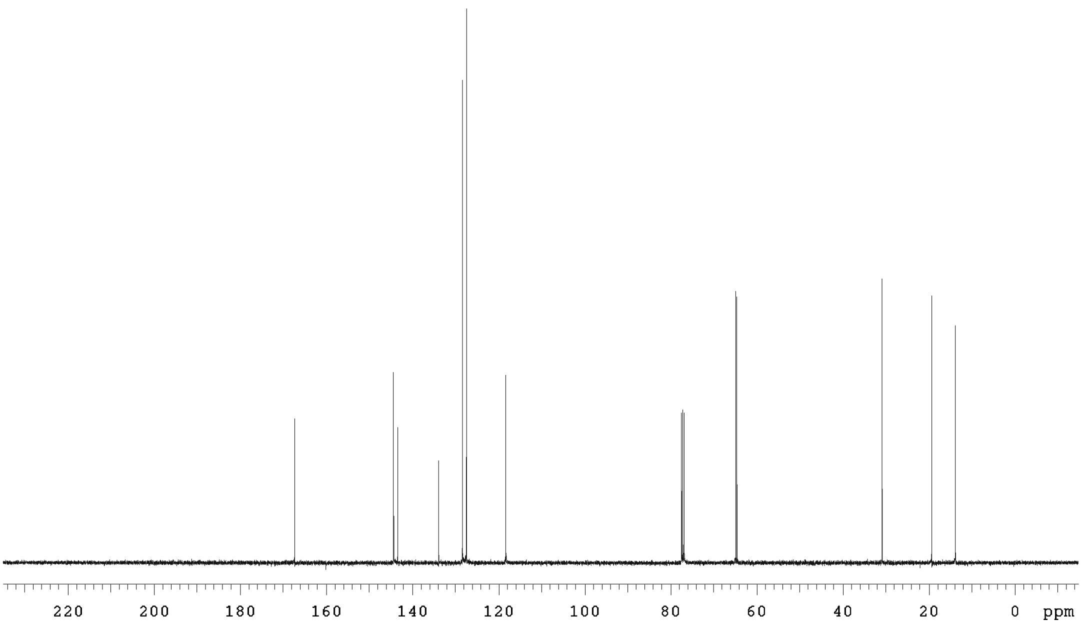
220
200
180
160
140
120
100
80
60
40
20
0
ppm

Chromatogram Plot

File: f:\jsox060_pure.sms
Sample: JSOx060_pure Operator: Org Farm Kemi
Scan Range: 1 - 1155 Time Range: 0.00 - 11.15 min. Date: 08/27/2009 09:16
Sample Notes: Routine

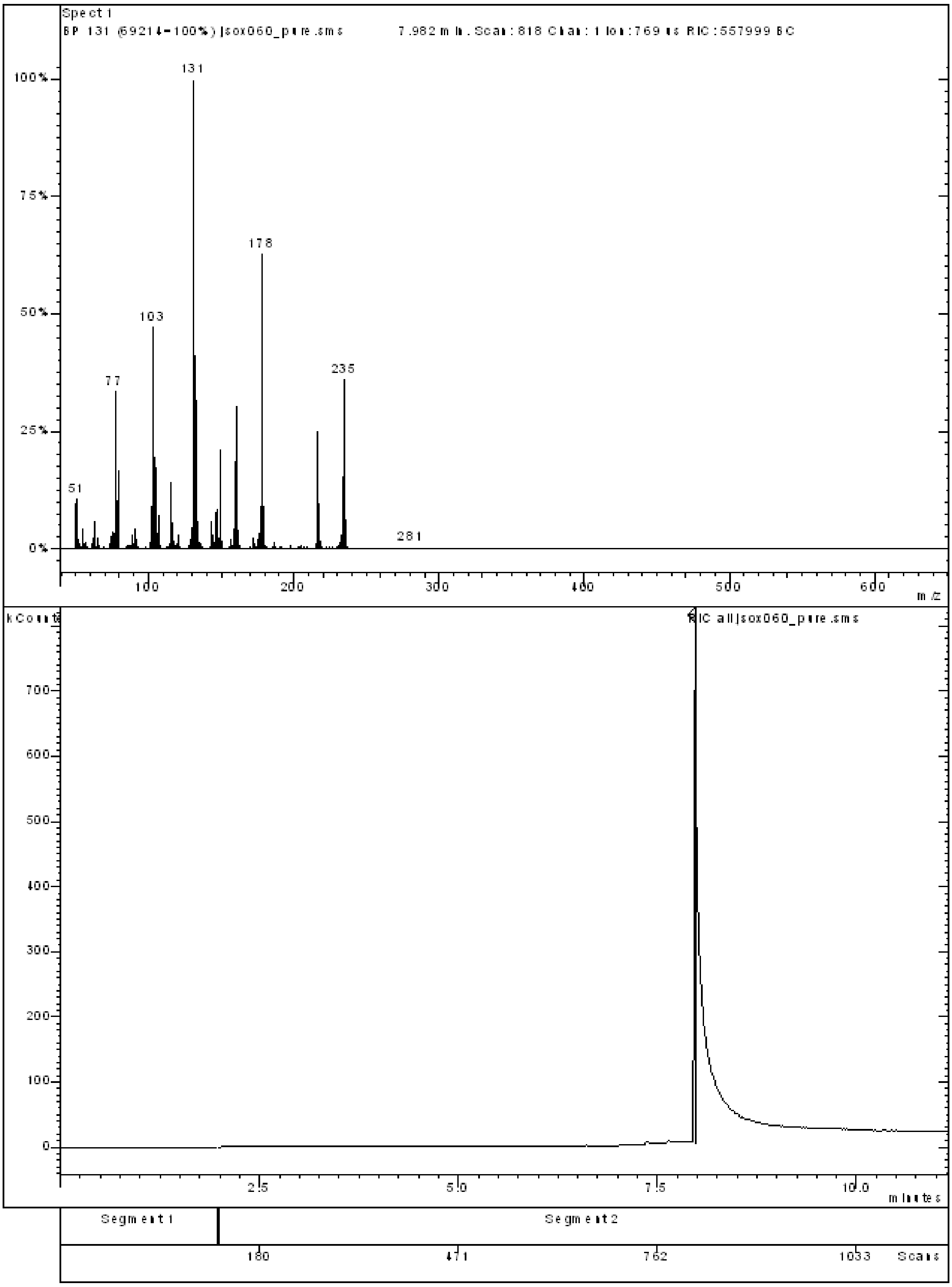

References

[1] E. J. Farrington, J. M. Brown, C. F. J. Barnard and E. Rowsell, *Angew. Chem. Int. Edit.* **2002**, *41*, 169-171.
[2] X. Y. Zhou, J. Y. Luo, J. Liu, S. M. Peng and G. J. Deng, *Org. Lett.* **2011**, *13*, 1432-1435.
[3] M. Z. Cai, J. Zhou, H. Zhao and C. S. Song, *J. Chem. Res-S.* **2002**, 76-78.
[4] M. Feuerstein, H. Doucet and M. Santelli, *J. Org. Chem.* **2001**, *66*, 5923-5925.
[5] M. Miura, H. Hashimoto, K. Itoh and M. Nomura, *J. Chem. Soc. Perk. T. 1* **1990**, 2207-2211.
[6] A. Maehara, H. Tsurugi, T. Satoh and M. Miura, *Org. Lett.* **2008**, *10*, 1159-1162.
[7] M. McConville, J. Blacker and J. L. Xiao, *Synthesis-Stuttgart* **2010**, 349-360.

^{1}H NMR, ^{13}C NMR and GC-MS spectra of compounds 2a-d, f-k

(*E*)-butyl-3-(4-methoxyphenyl)acrylate (2a)

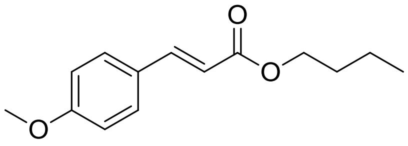

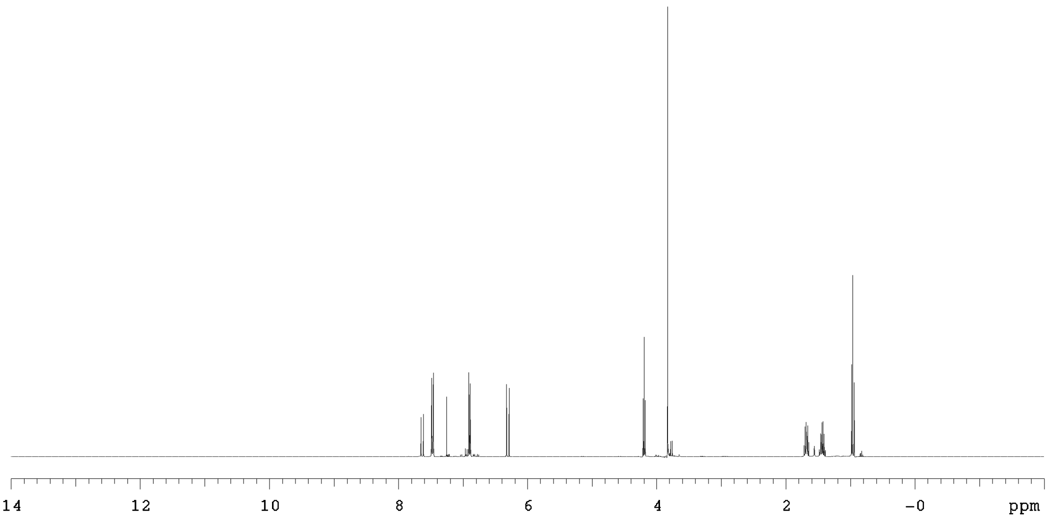

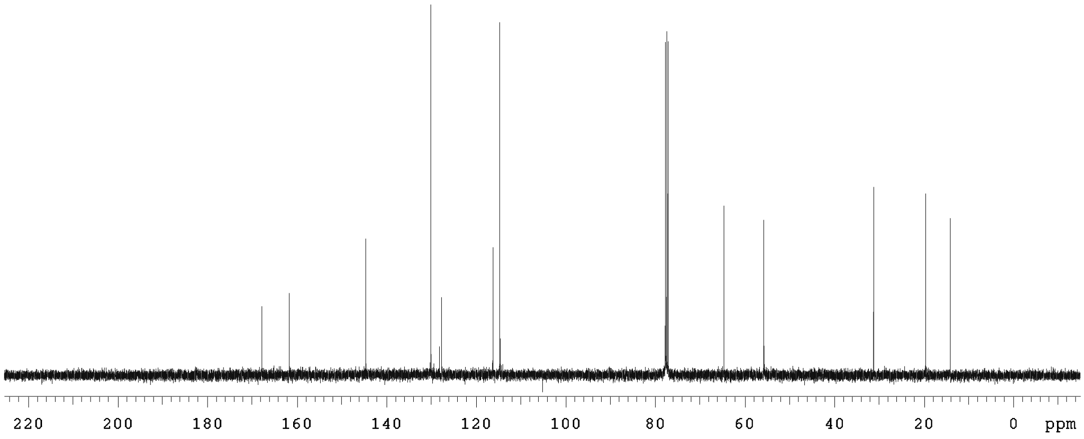

Chromatogram Plot

File: f:\jsox016.sms
Sample: JSOx016
Scan Range: 1 - 1282 Time Range: 0.00 - 12.98 min.
Sample Notes: Routine

Operator: Org Farm Kemi
Date: 08/19/2009 11:44

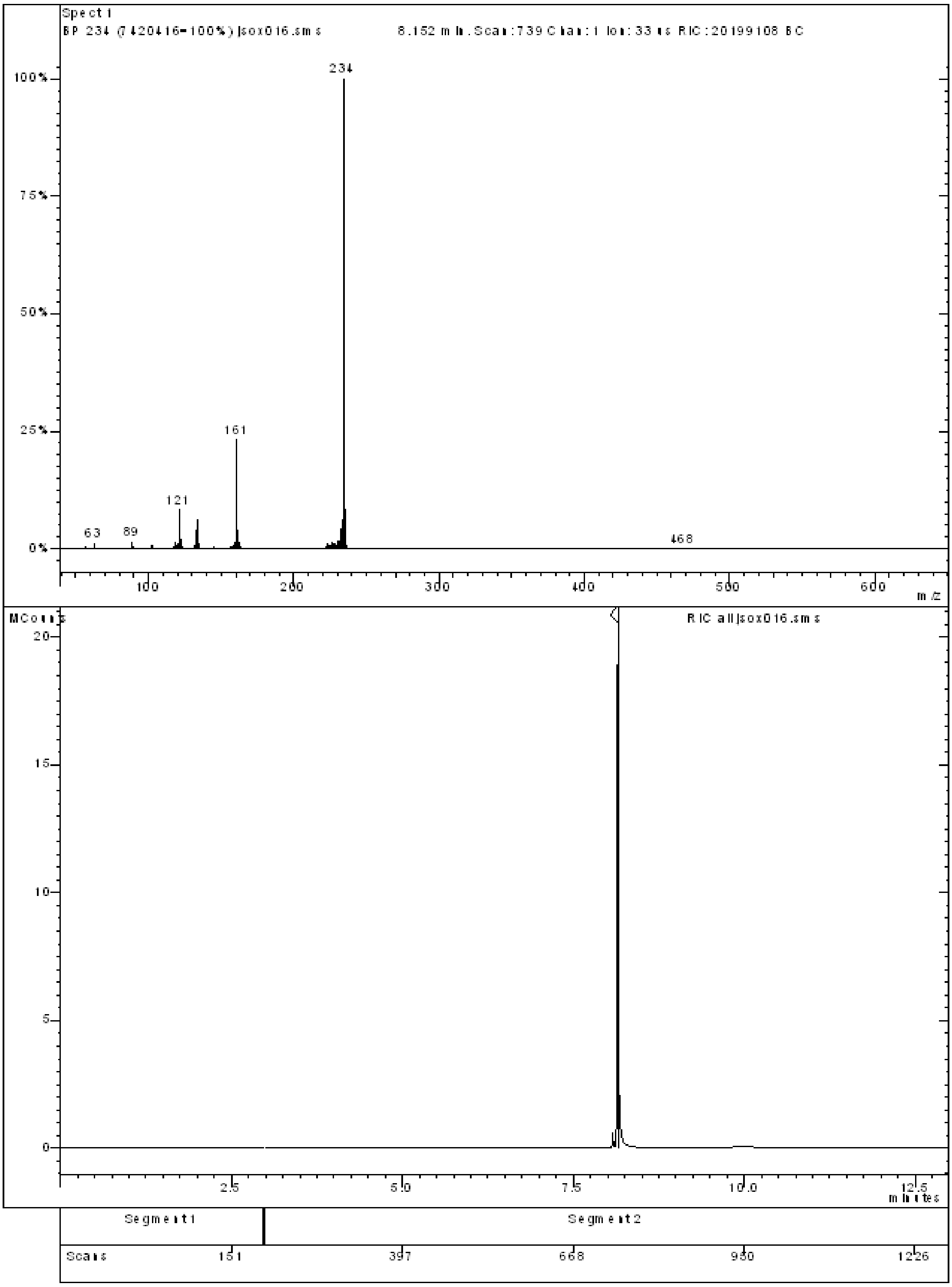

(*E*)-butyl 3-(4-*tert*-butylphenyl)acrylate (2b)

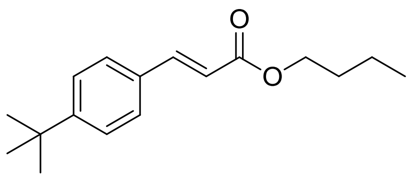

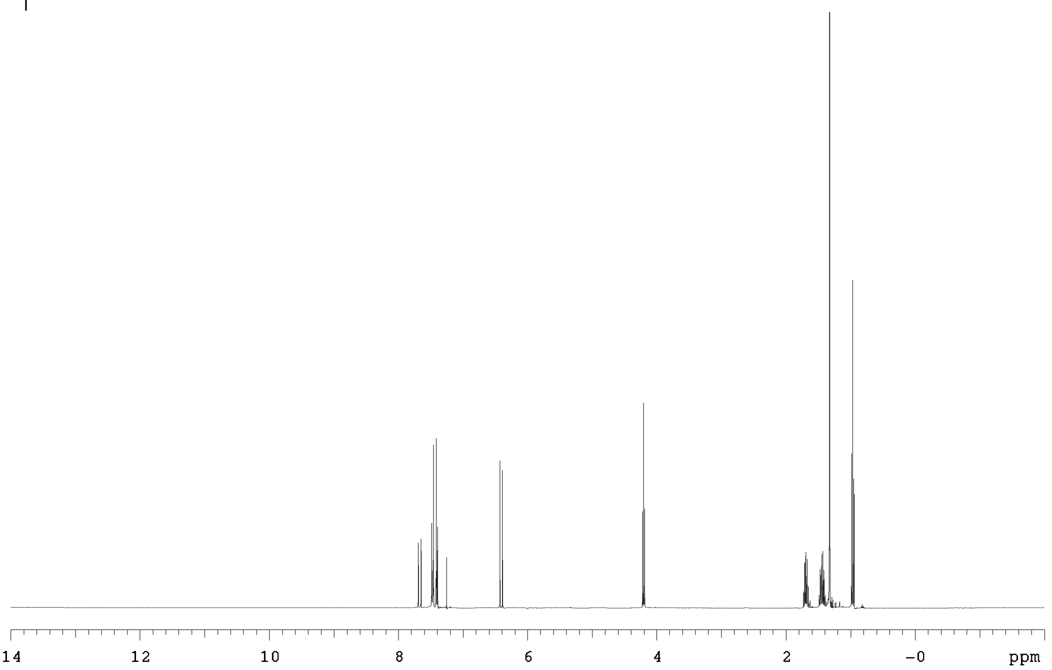

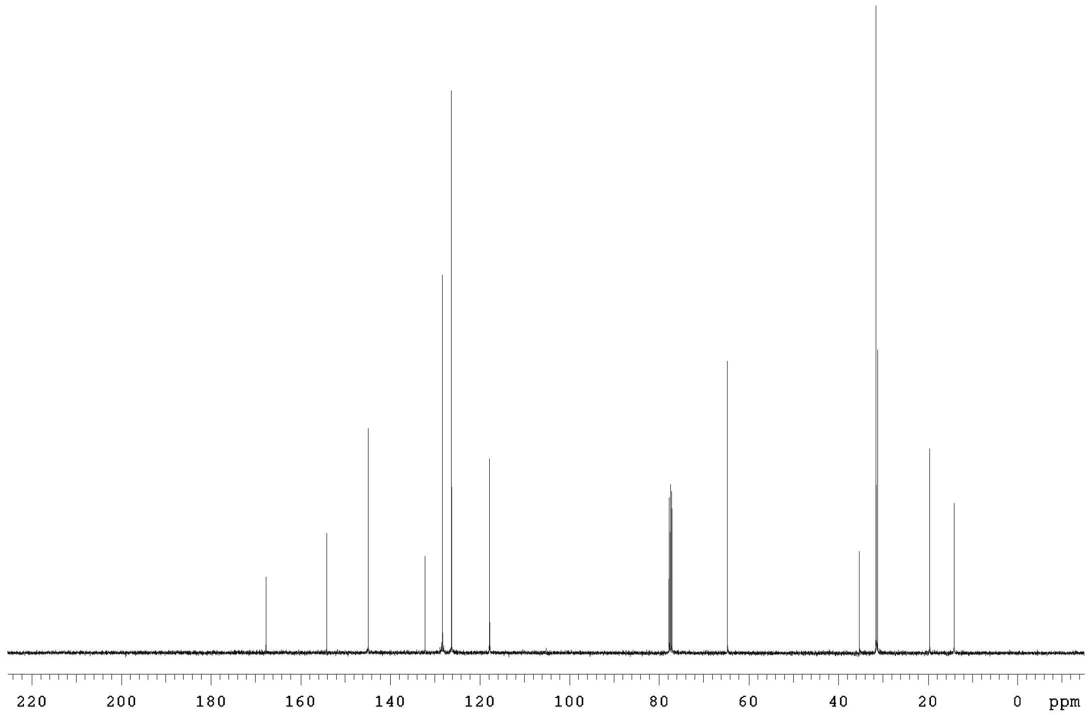

Chromatogram Plot

File: f:\jsox011_pure.sms
Sample: JSOx011_pure
Scan Range: 1 - 1267 Time Range: 0.00 - 12.99 min.
Sample Notes: Routine

Operator: Org Farm Kemi
Date: 08/24/2009 14:04

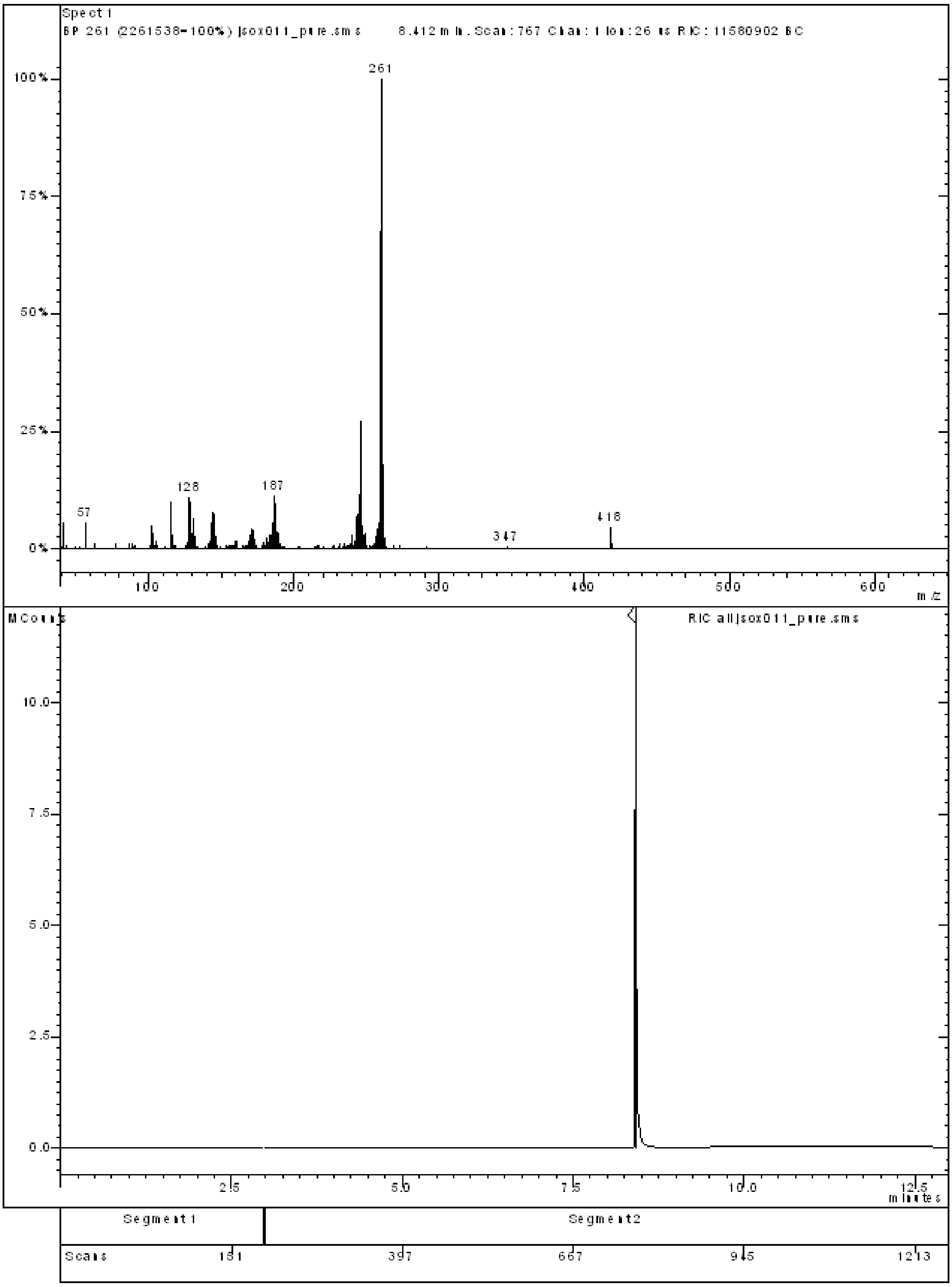

(*E*)-butyl-3-*p*-tolylacrylate (2c)

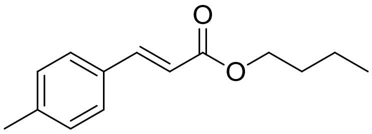

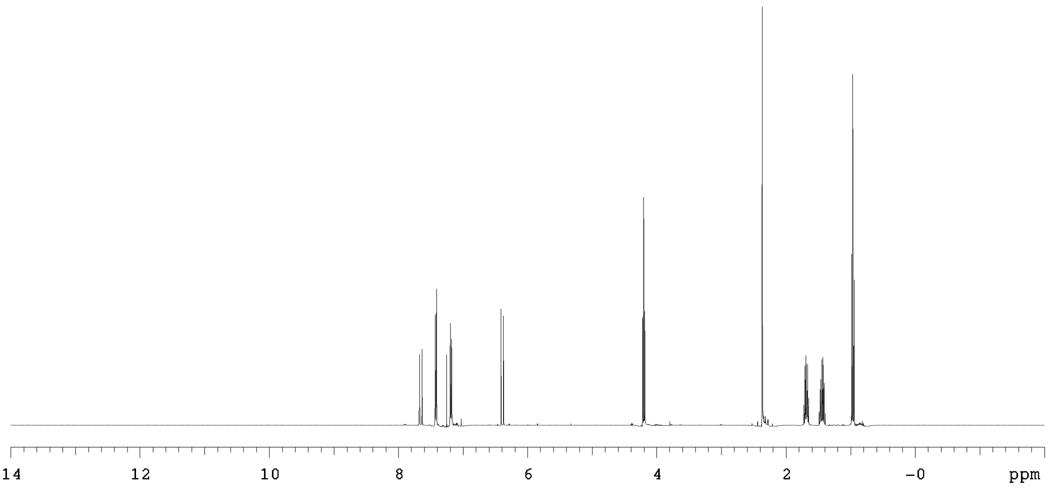

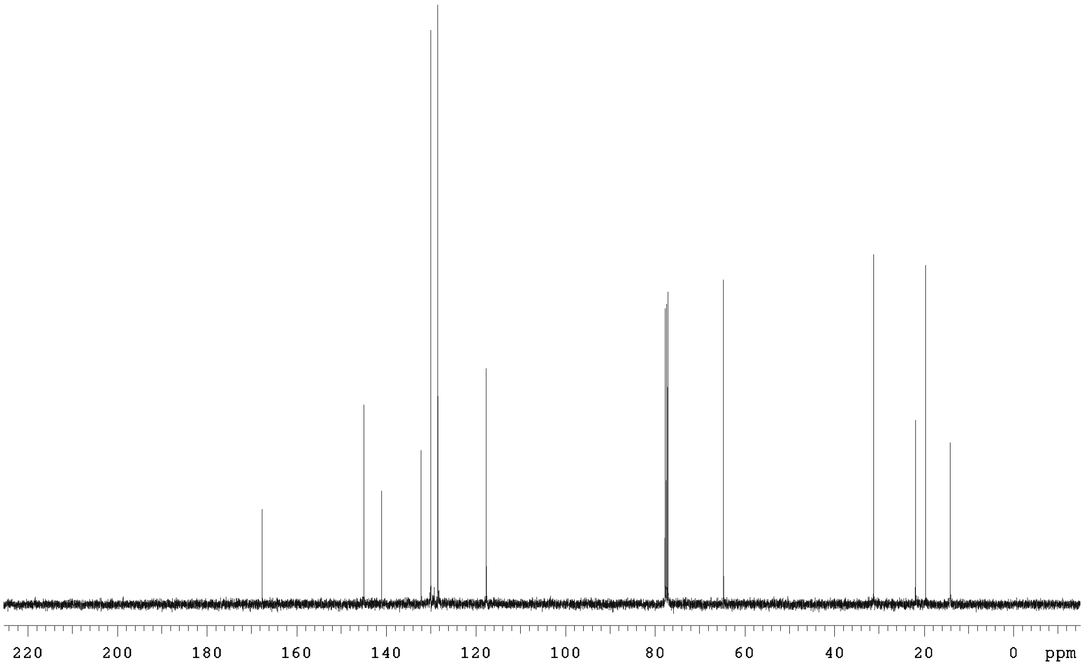

Chromatogram Plot

File: f:\jsox007.sms
Sample: JSOx007 Operator: Org Farm Kemi
Scan Range: 1 - 1291 Time Range: 0.00 - 12.98 min. Date: 08/19/2009 12:50
Sample Notes: Routine

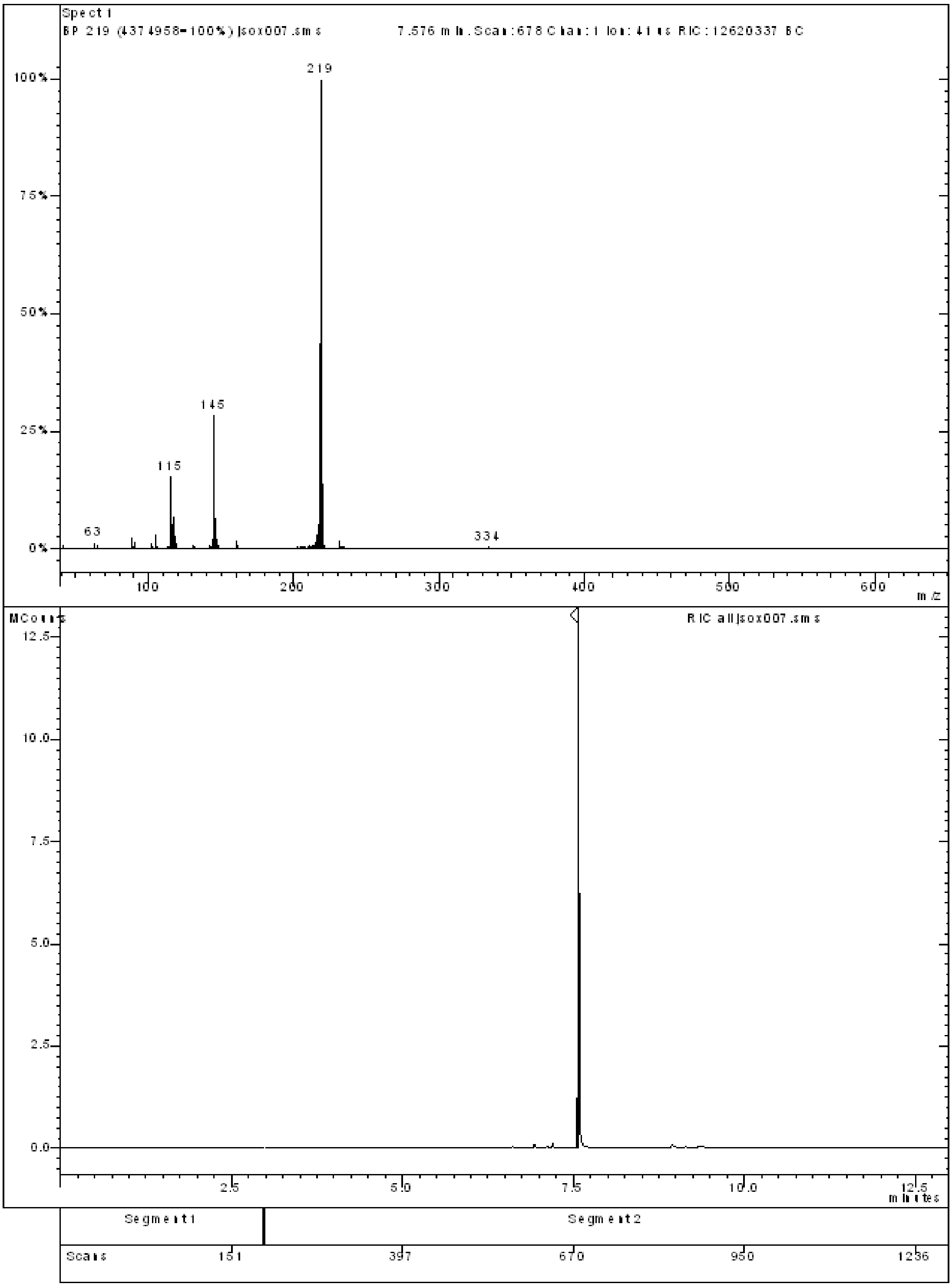

(*E*)-butyl cinnamate (2d)

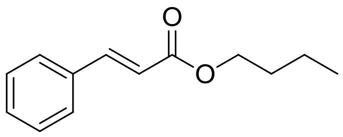

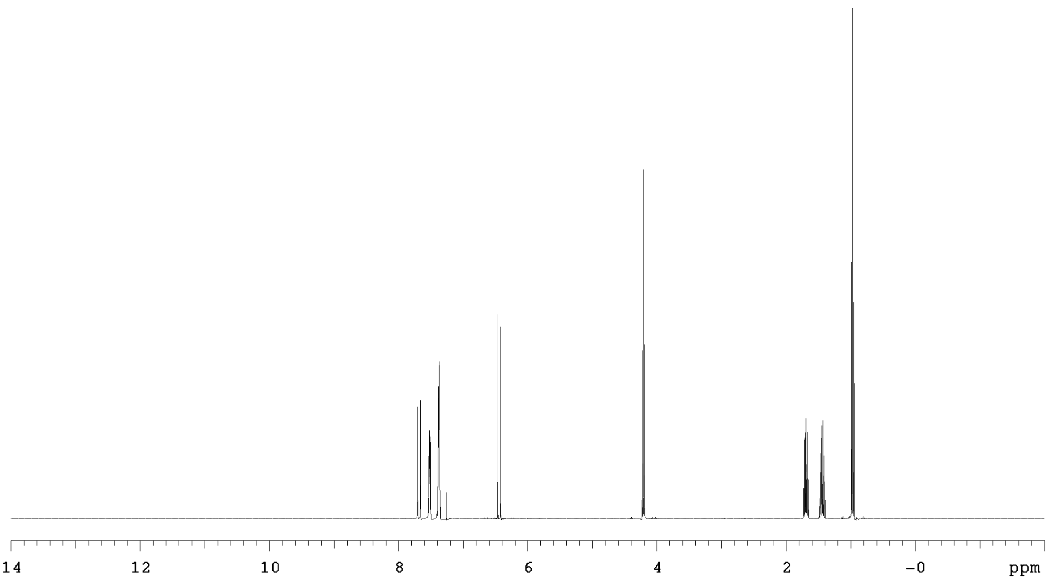

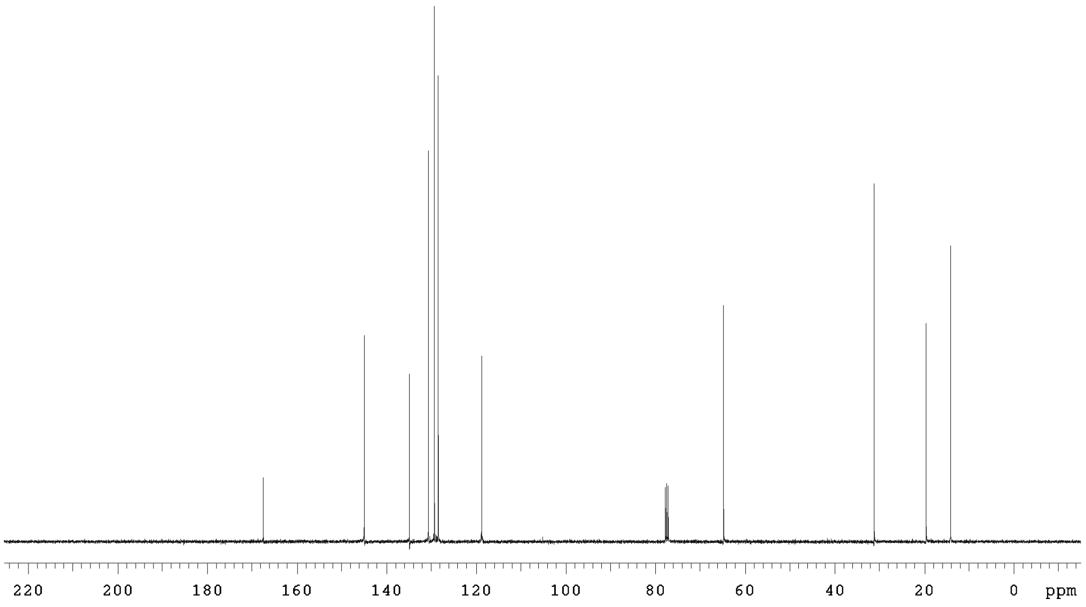

Chromatogram Plot

File: f:\jsox022_pure.sms
Sample: JSOx022_pure
Scan Range: 1 - 1295 Time Range: 0.00 - 12.99 min.
Sample Notes: Routine

Operator: Org Farm Kemi
Date: 08/19/2009 14:09

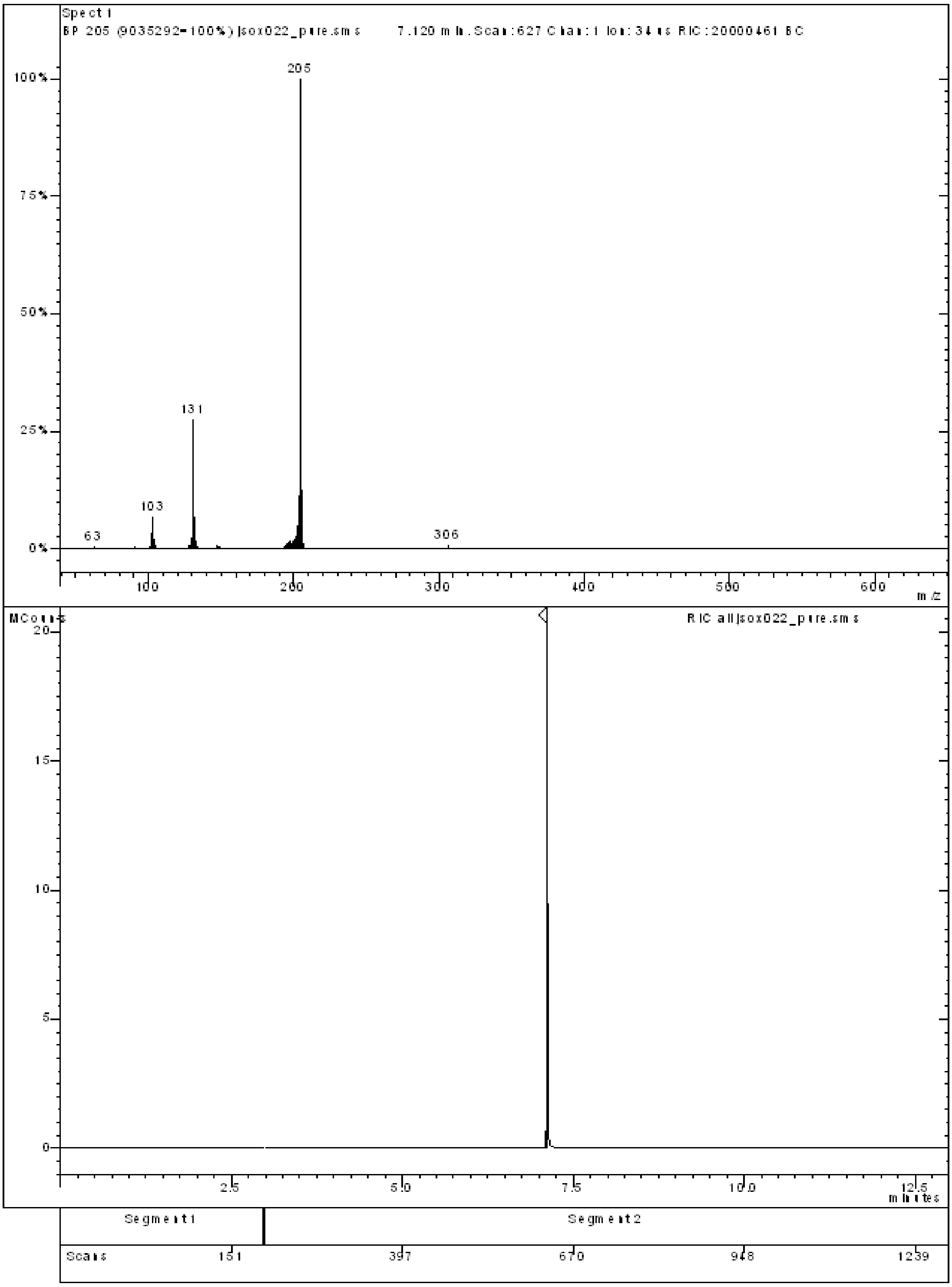

(*E*)-butyl 3-(4-bromophenyl)acrylate (2f)

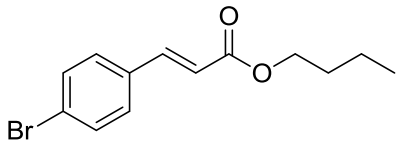

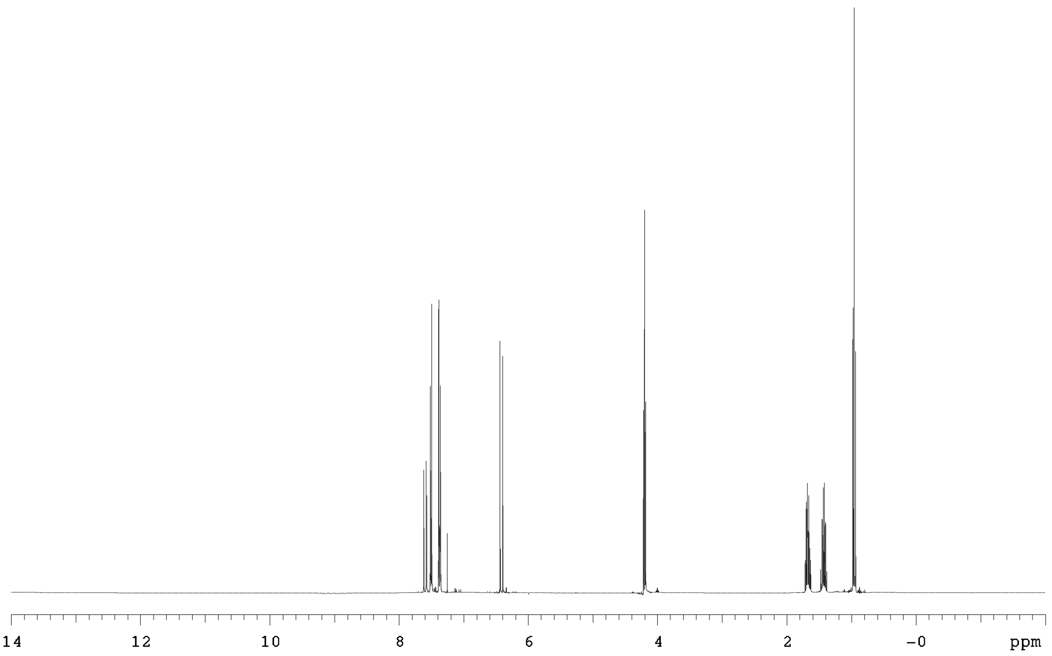

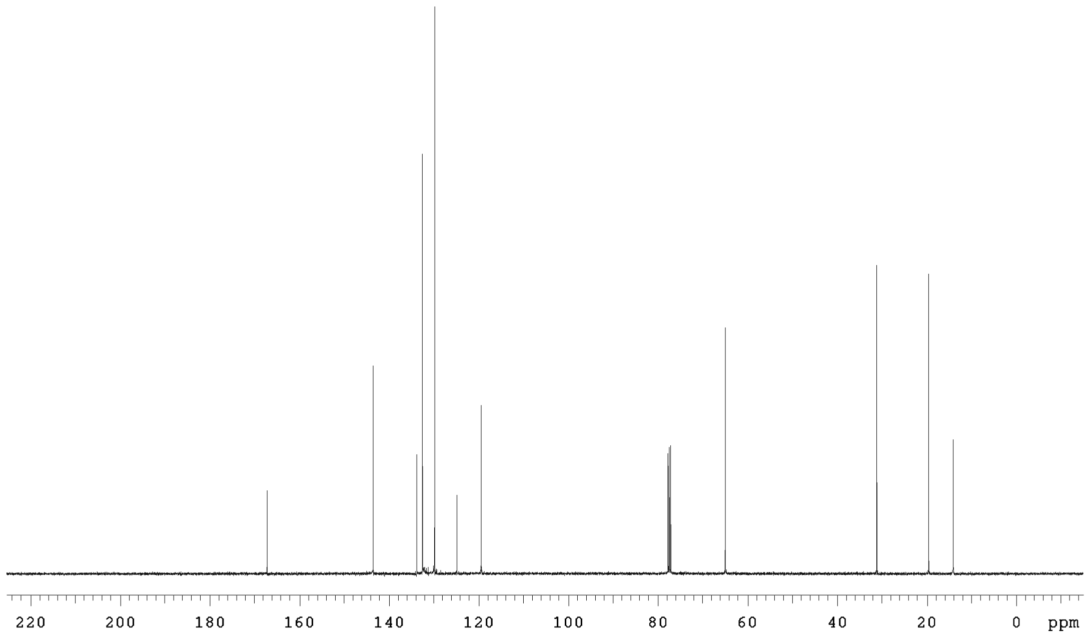

Chromatogram Plot

File: f:\jsox017_pure.sms
Sample: JSOx017_pure
Operator: Org Farm Kemi
Scan Range: 1 - 1172 Time Range: 0.00 - 11.16 min.
Date: 08/28/2009 12:04
Sample Notes: Routine

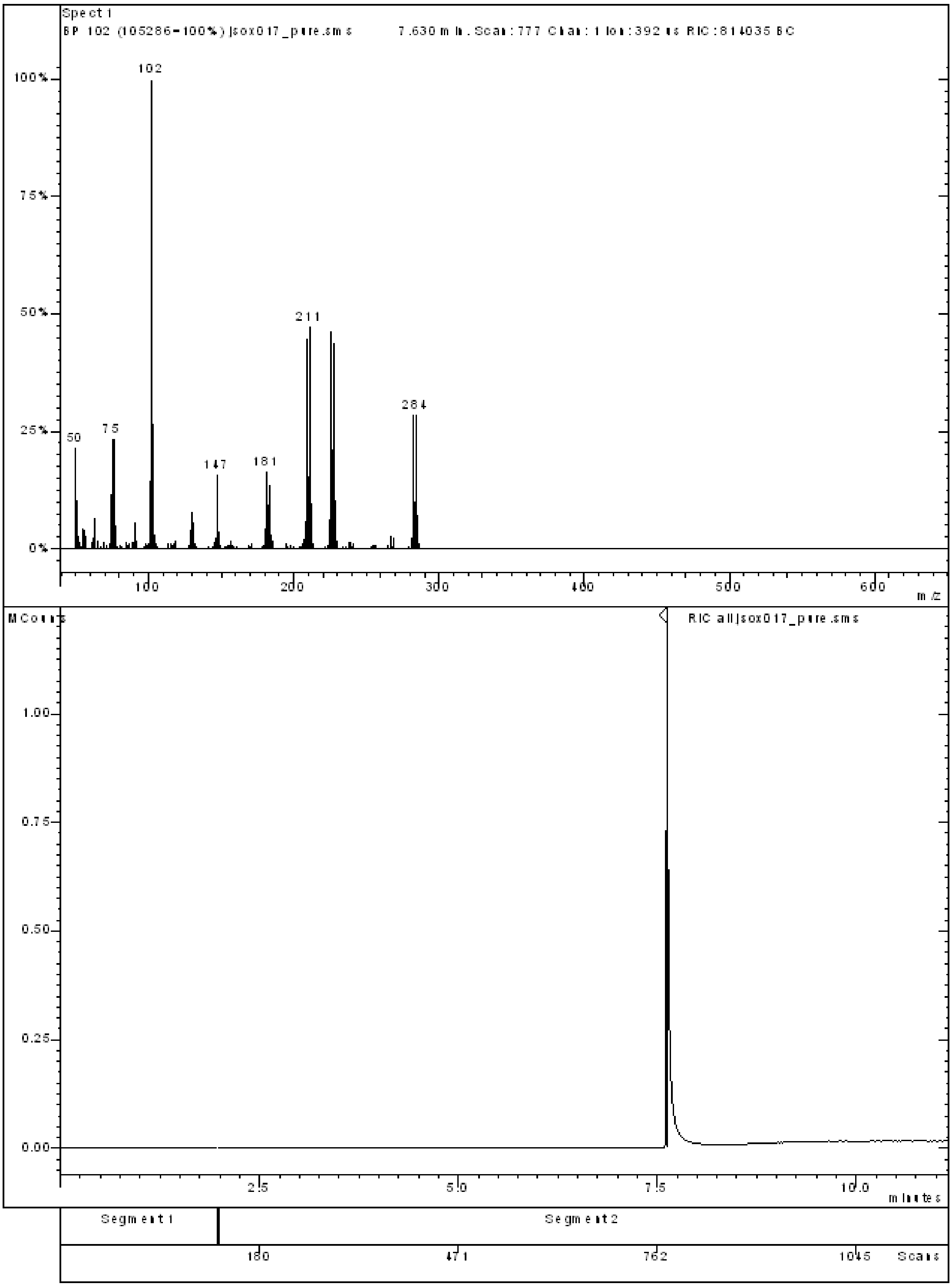

(*E*)-butyl 3-(4-acetylphenyl)acrylate (2g)

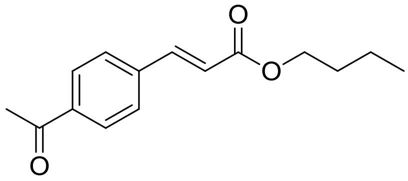

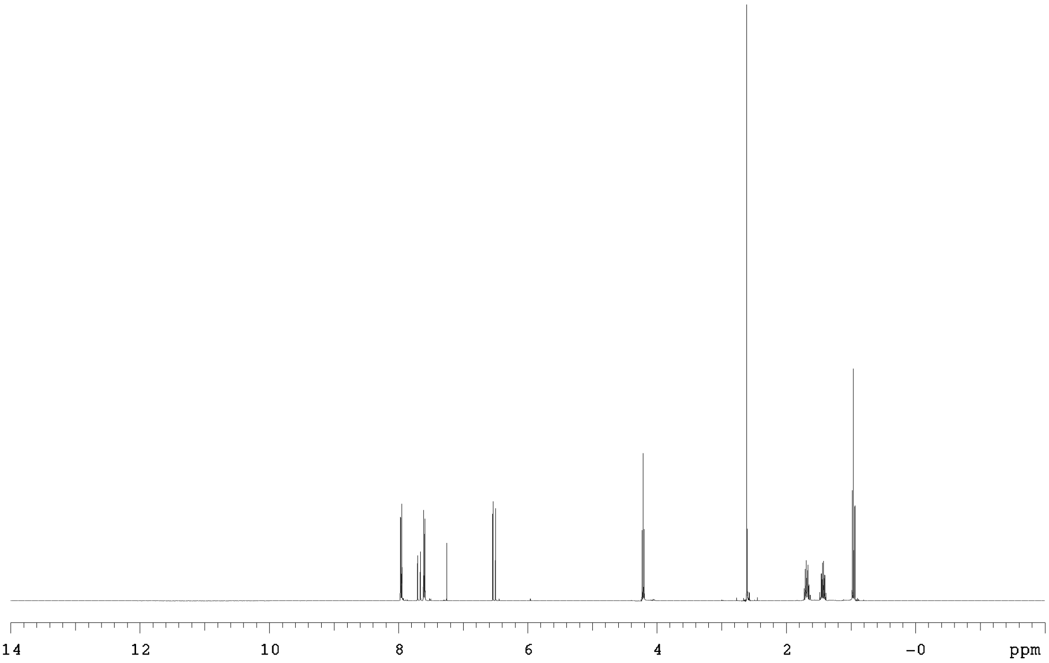

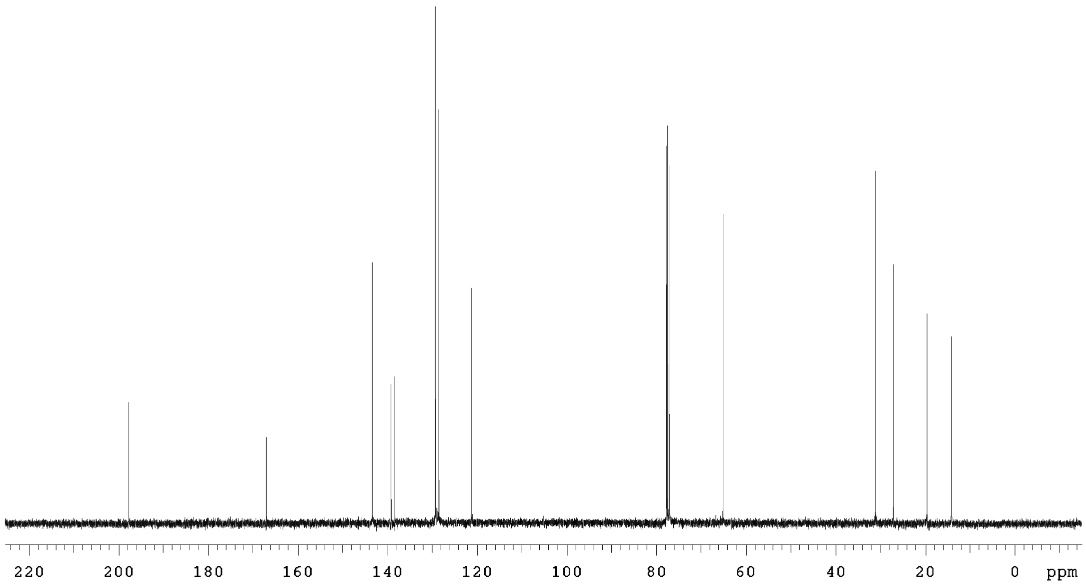

Chromatogram Plot

File: f:\jsox019.sms
Sample: JSOx019
Scan Range: 1 - 1259 Time Range: 0.00 - 12.99 min.
Sample Notes: Routine

Operator: Org Farm Kemi
Date: 08/24/2009 11:08

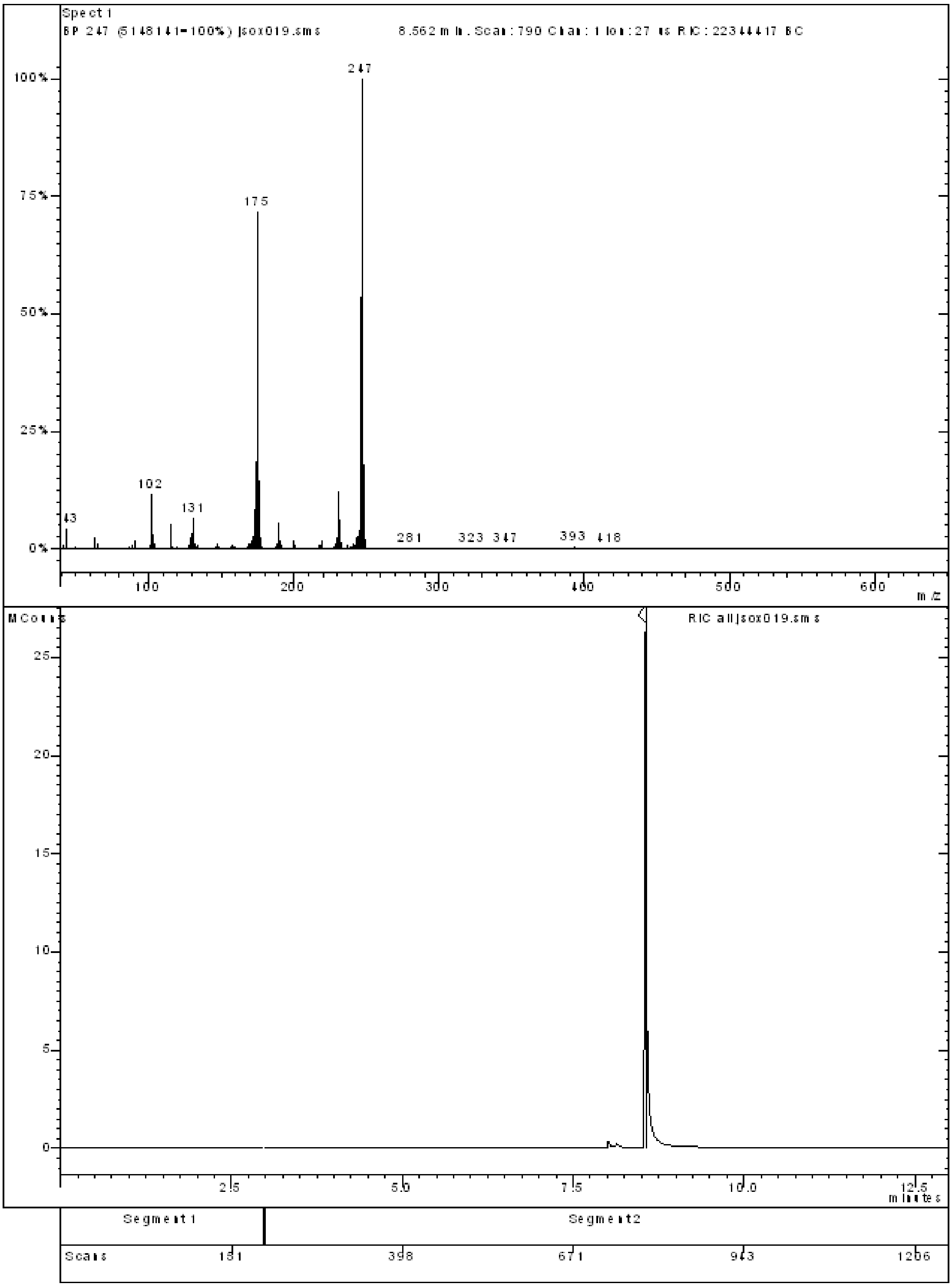

(*E*)-butyl-3-(4-(trifluoromethyl)phenyl)acrylate (2h)

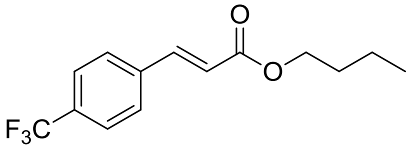

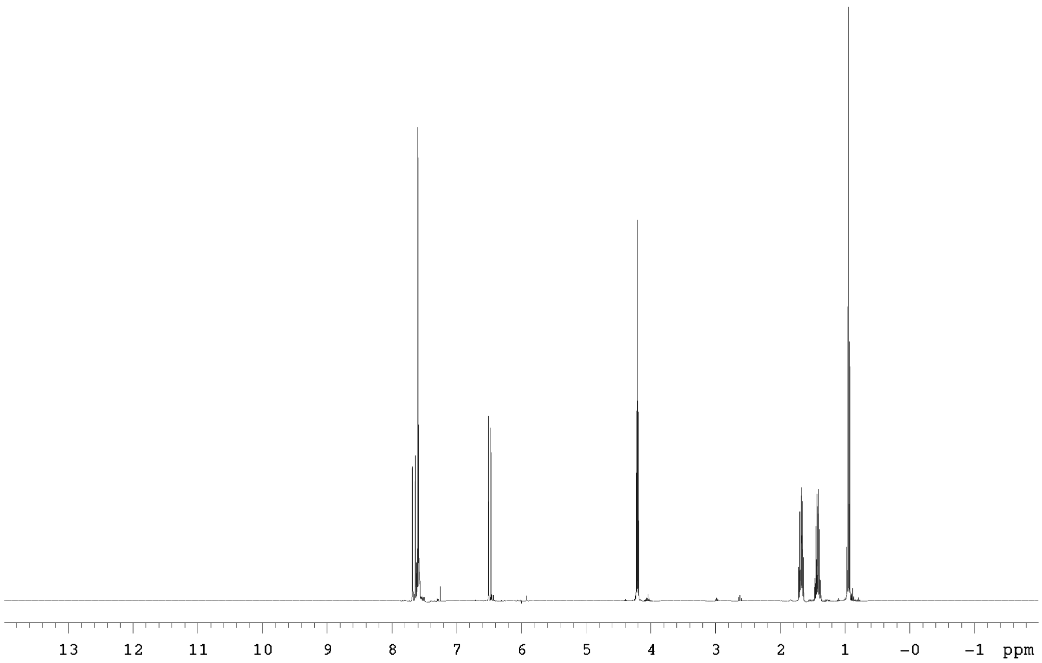

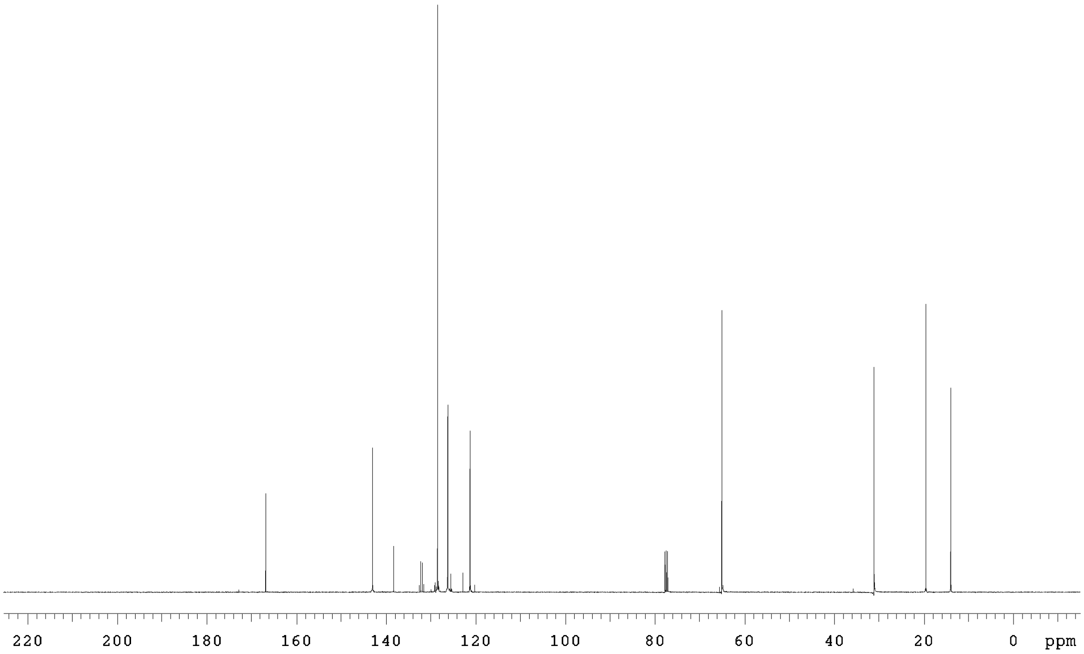

Chromatogram Plot

File: f:\jsox013.sms
Sample: JSOx013 Operator: Org Farm Kemi
Scan Range: 1 - 1510 Time Range: 0.00 - 14.98 min. Date: 01/22/2009 15:08
Sample Notes: Today

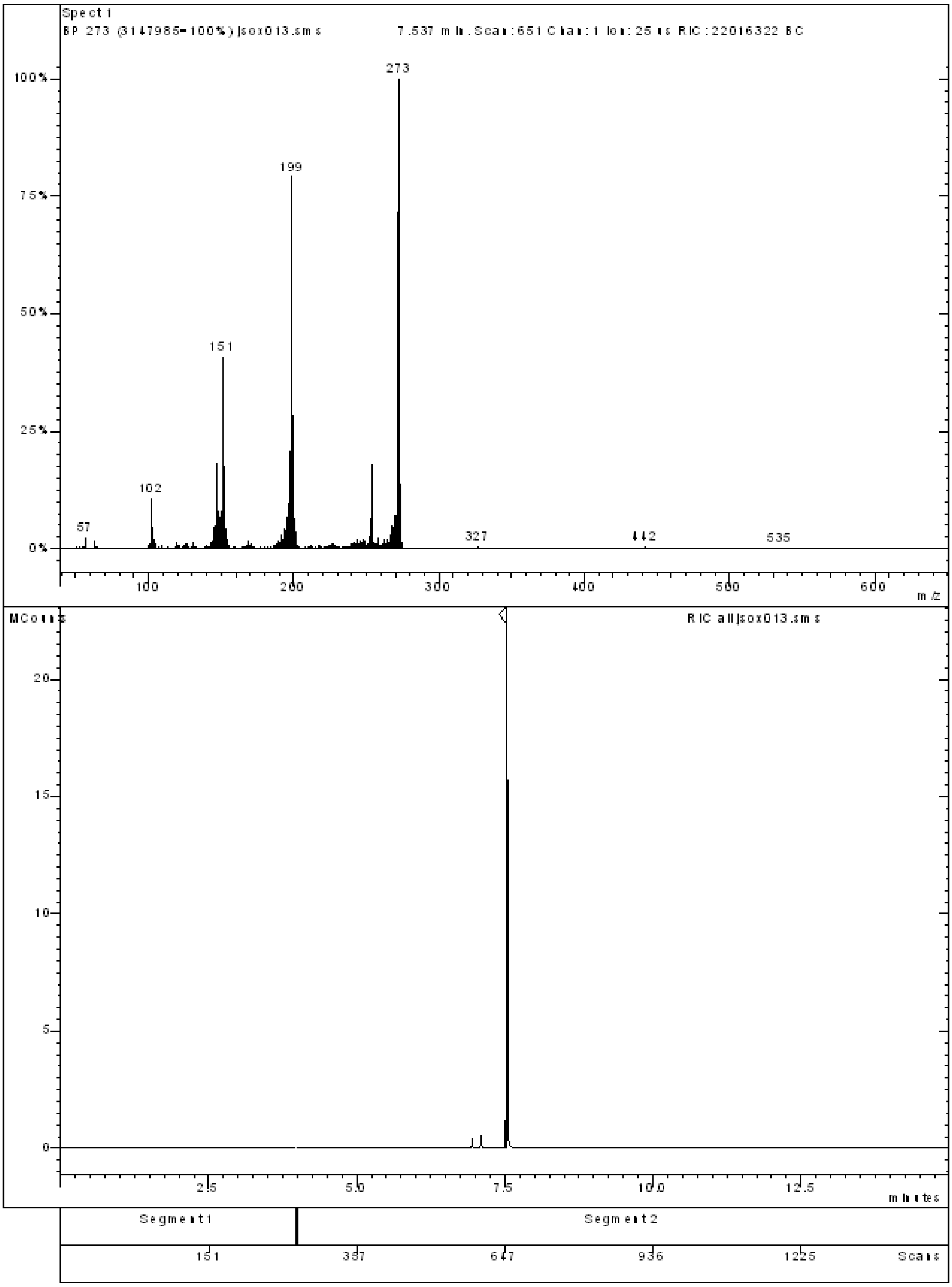

(*E*)-butyl 3-o-tolylacrylate (2i)

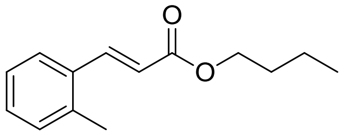

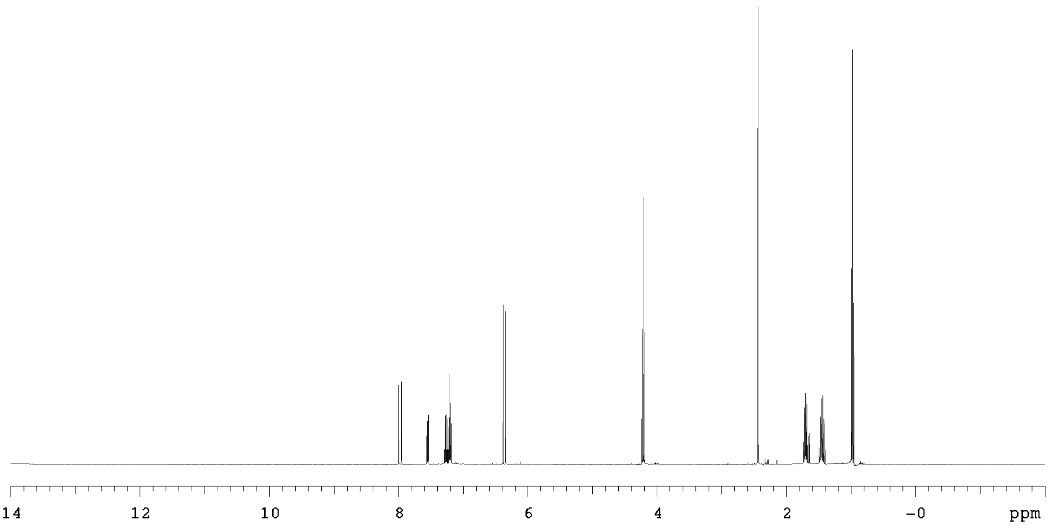

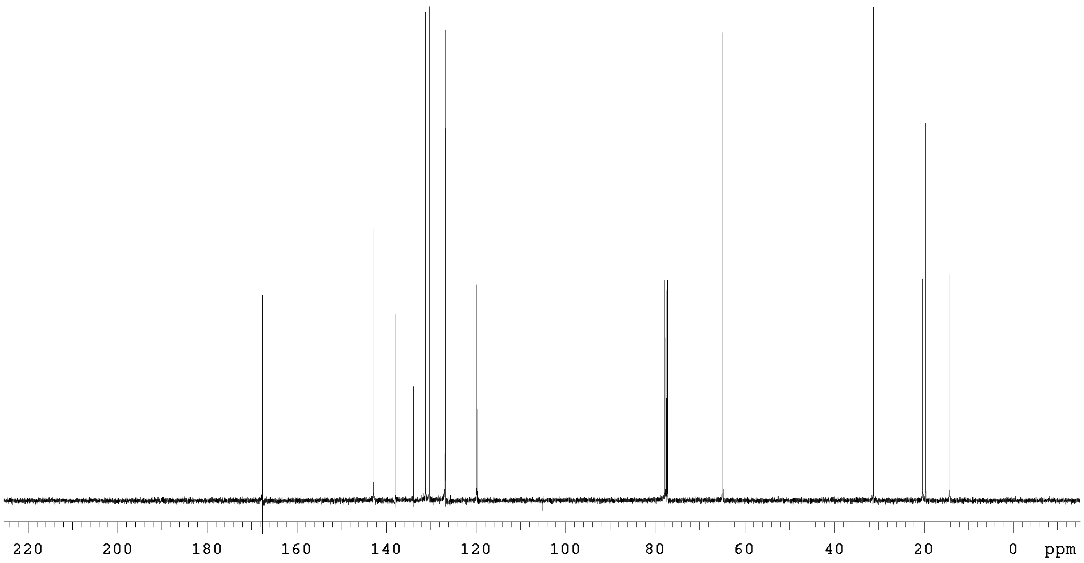

Chromatogram Plot

File: g:\supportingox\jsox025_pure.sms
Sample: JSOx025_pure
Operator: Org Farm Kemi
Scan Range: 1 - 1176 Time Range: 0.00 - 11.15 min.
Date: 09/02/2009 09:30
Sample Notes: Routine

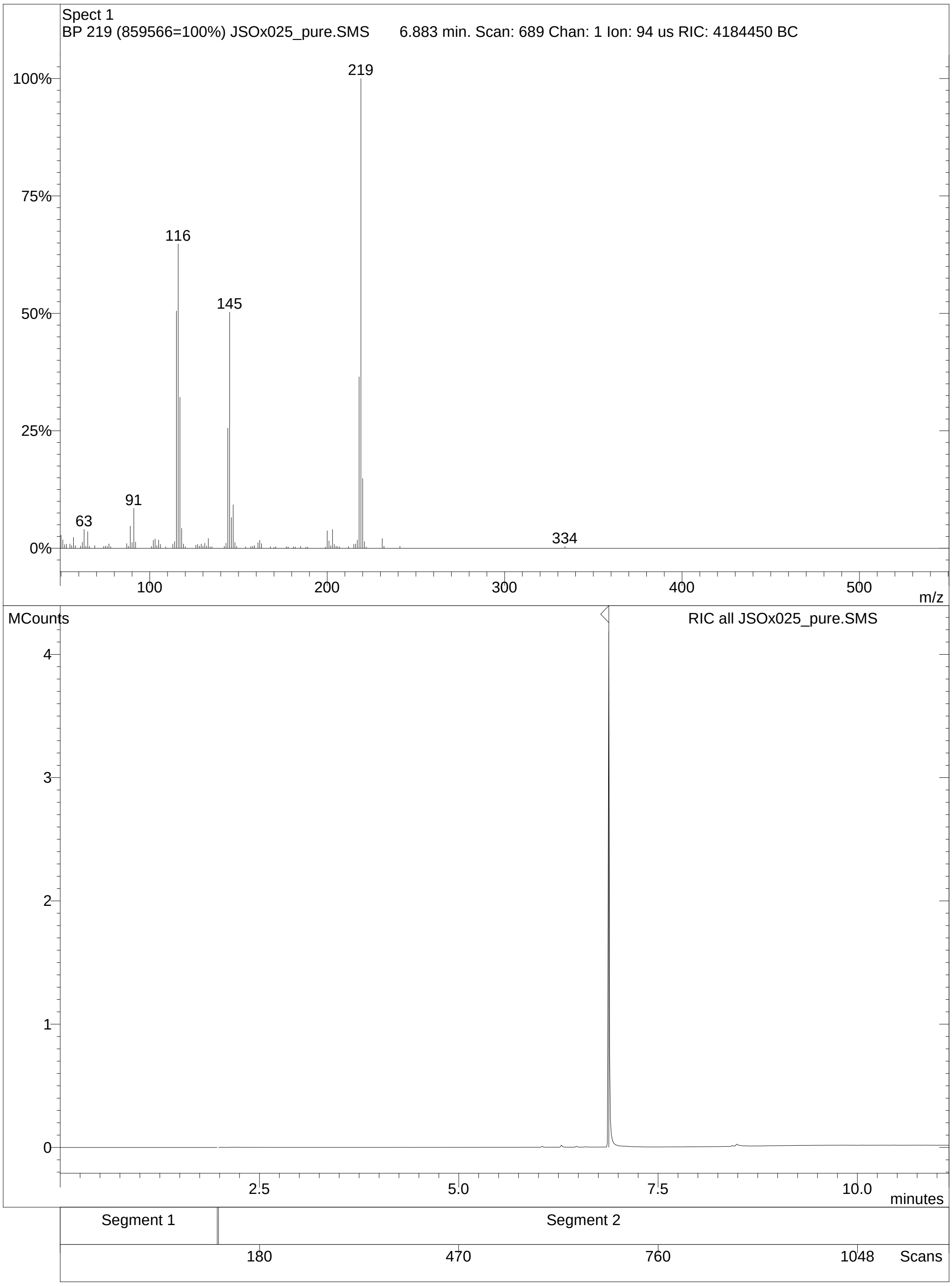

(*E*)-butyl 3-(naphthalen-2-yl)acrylate (2j)

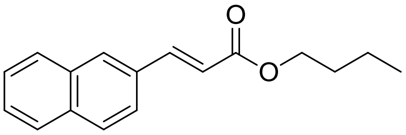

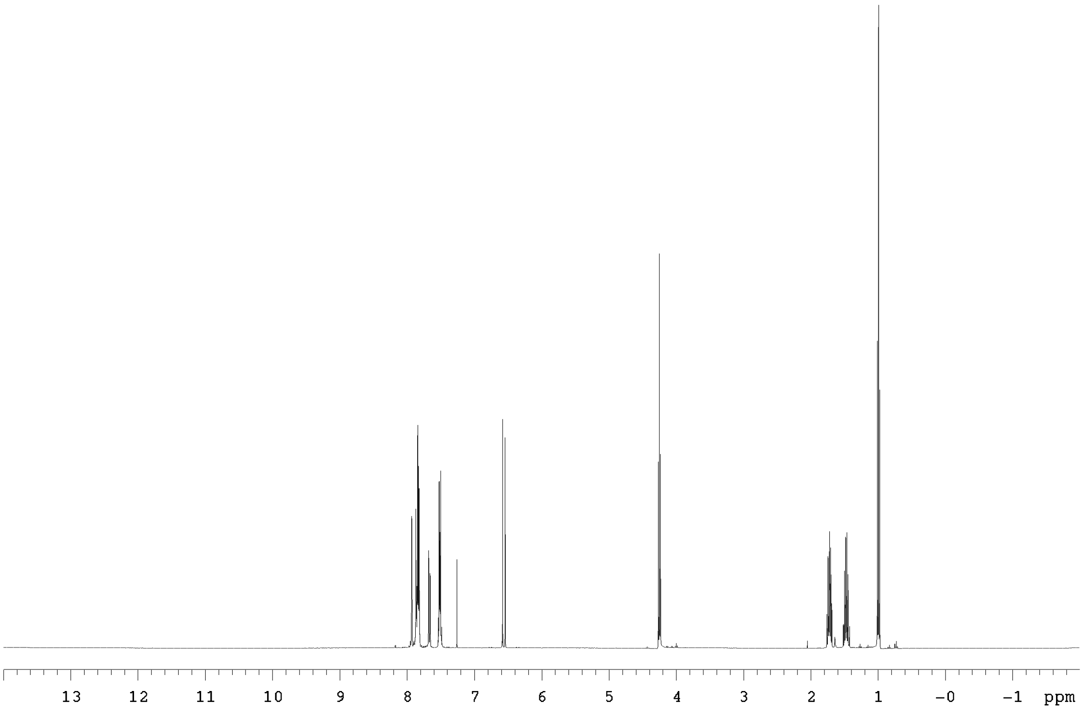

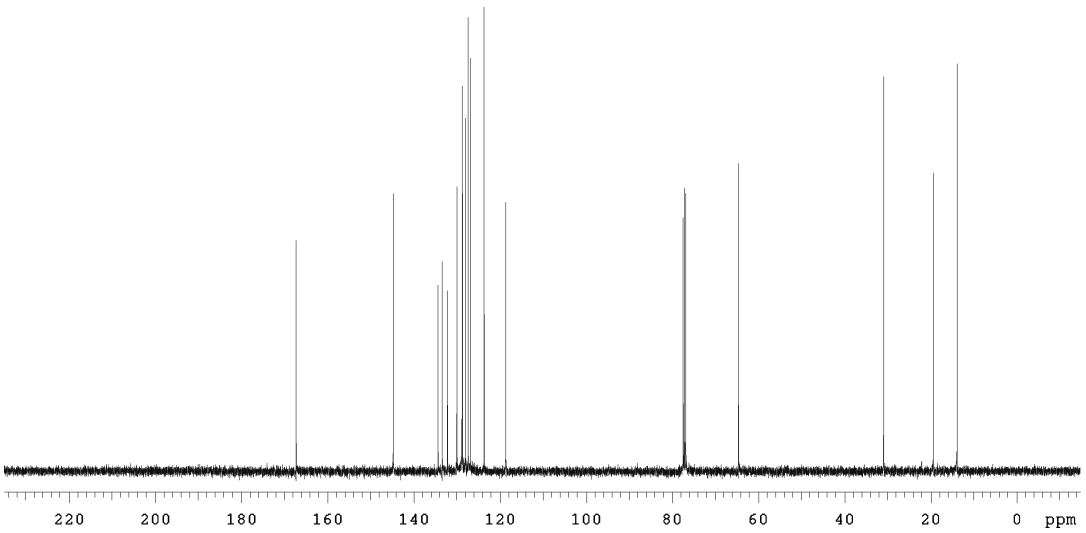

Chromatogram Plot

File: g:\gc-ms\jsox5057_.sms
Sample: JSOx5057_ Operator: Org Farm Kemi
Scan Range: 1 - 1184 Time Range: 0.00 - 11.15 min. Date: 10/18/2011 16:18
Sample Notes: Routine

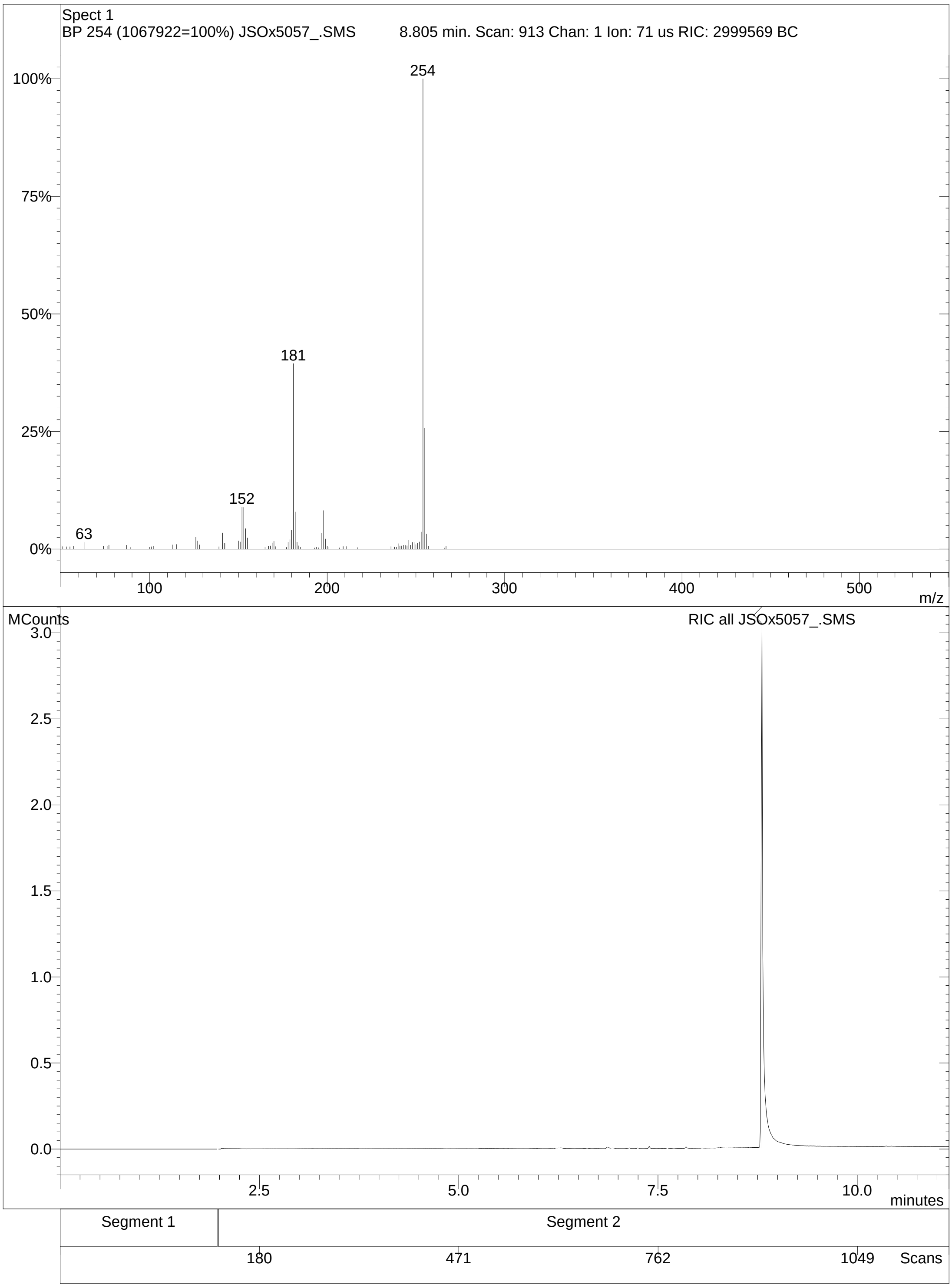

(*E*)-butyl 3-(furan-2-yl)acrylate (2k)

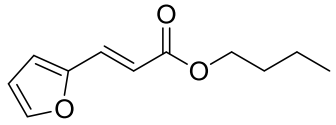

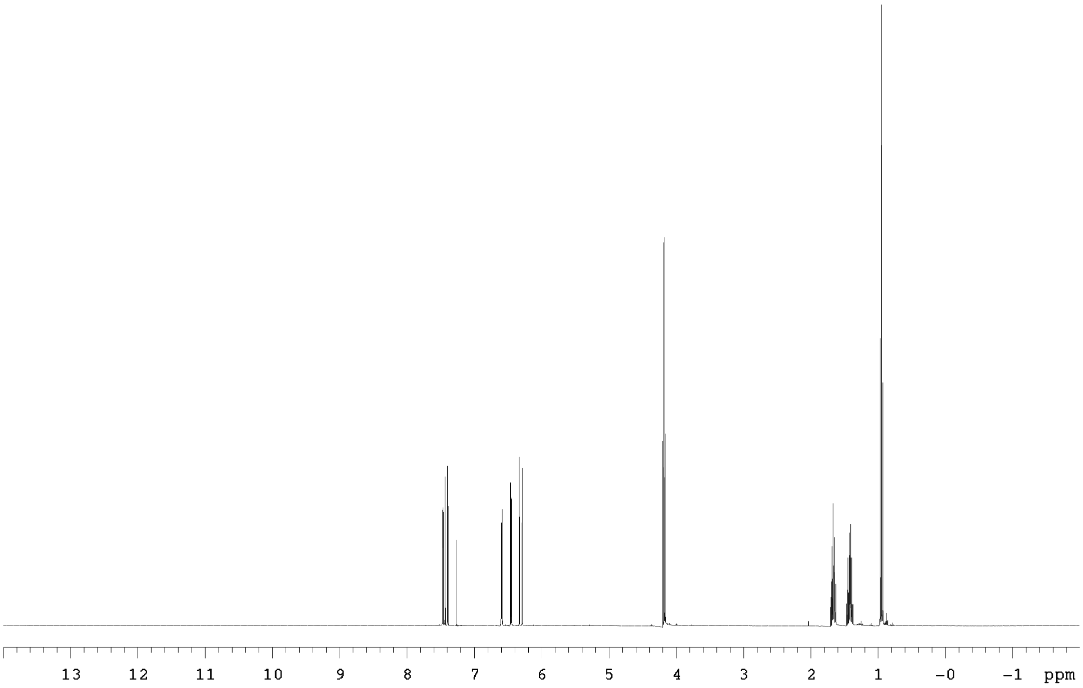

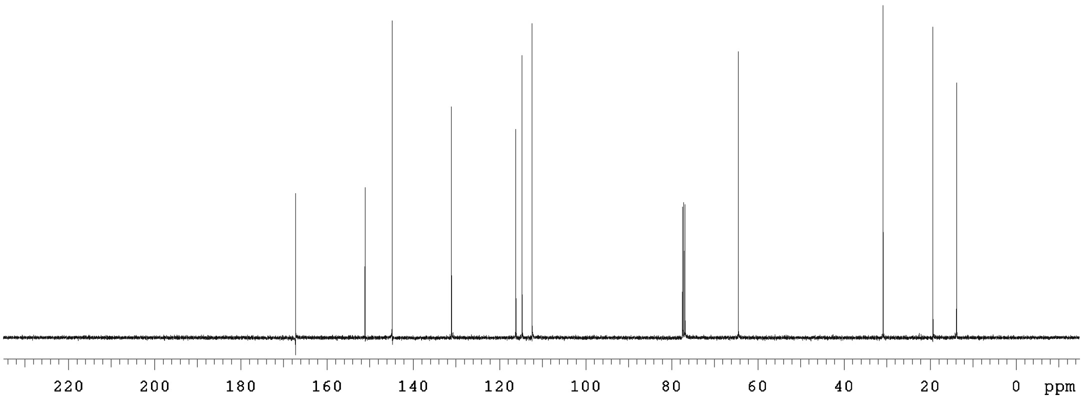

Chromatogram Plot

File: g:\gc-ms\jsox5021_.sms
Sample: JSOx5021_f9
Scan Range: 1 - 1127 Time Range: 0.00 - 11.15 min.
Sample Notes: Routine

Operator: Org Farm Kemi
Date: 10/19/2011 12:57

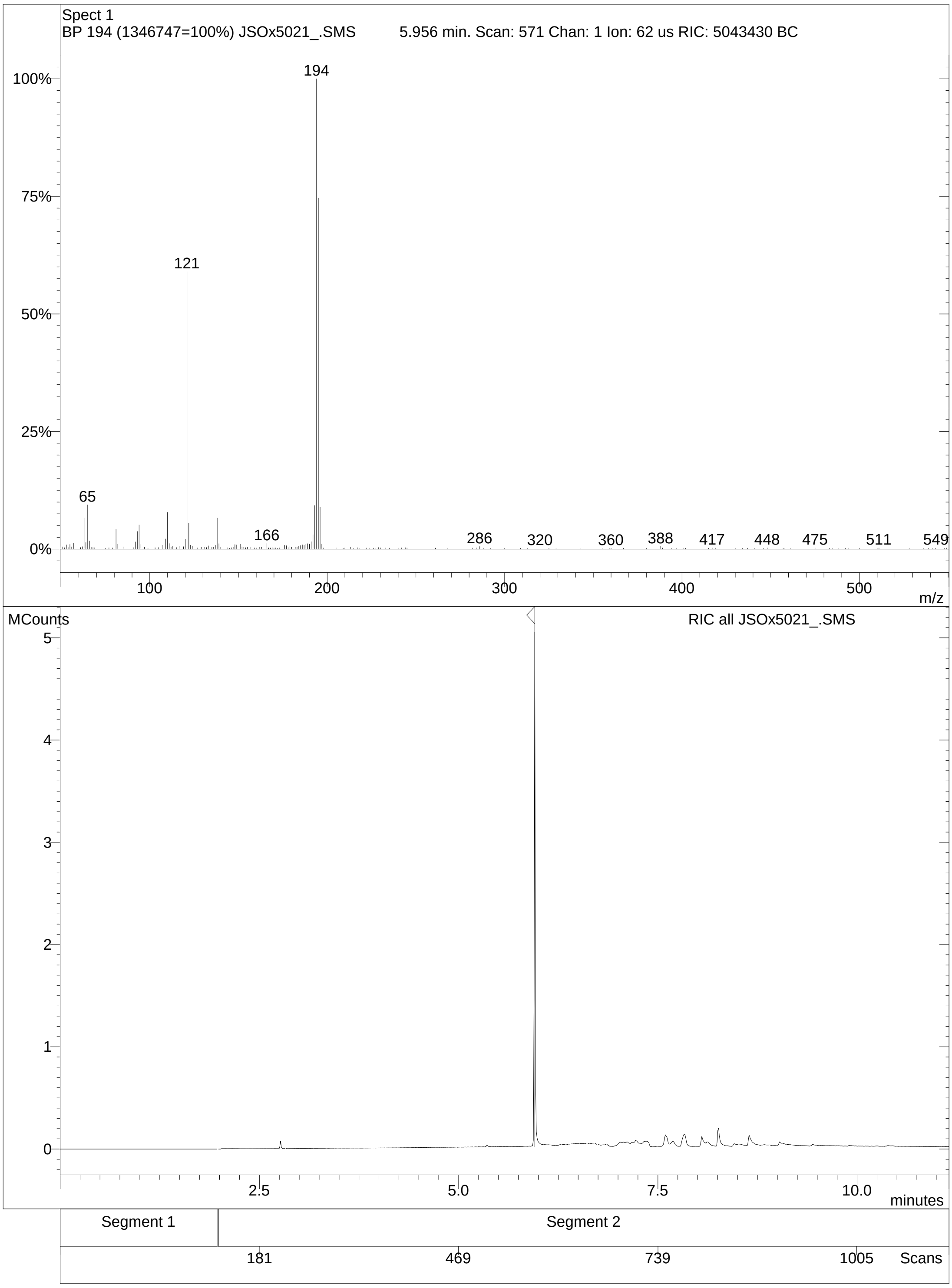

GC-MS spectra of compounds 3a and 4a-f

1-(4-methoxyphenyl)ethanone (4a)

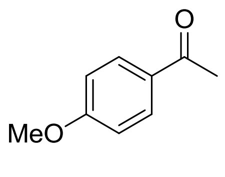

Chromatogram Plot

File: g:\gc-ms\jsox5059_.sms
Sample: JSOx5059_
Scan Range: 1 - 1169 Time Range: 0.00 - 11.15 min.
Sample Notes: Routine

Operator: Org Farm Kemi
Date: 10/24/2011 16:02

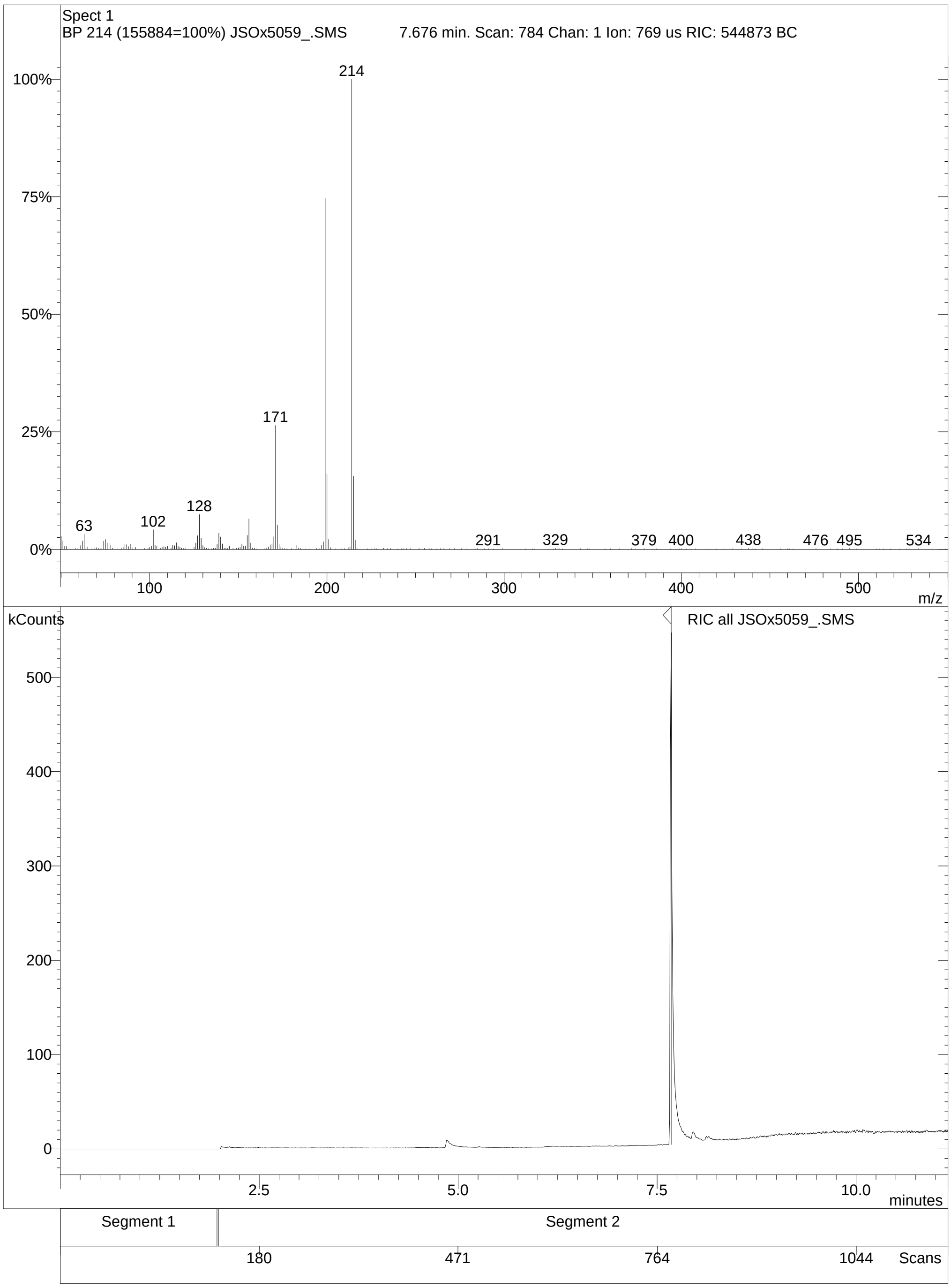

1-(4-(tert-butyl)phenyl)ethanone (4b)

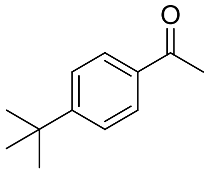

Chromatogram Plot

File: g:\gc-ms\jsox041_pure.sms
Sample: jsox041_pure
Scan Range: 1 - 1304 Time Range: 0.00 - 12.98 min.
Sample Notes: Routine

Operator: Org Farm Kemi
Date: 01/29/2009 17:04

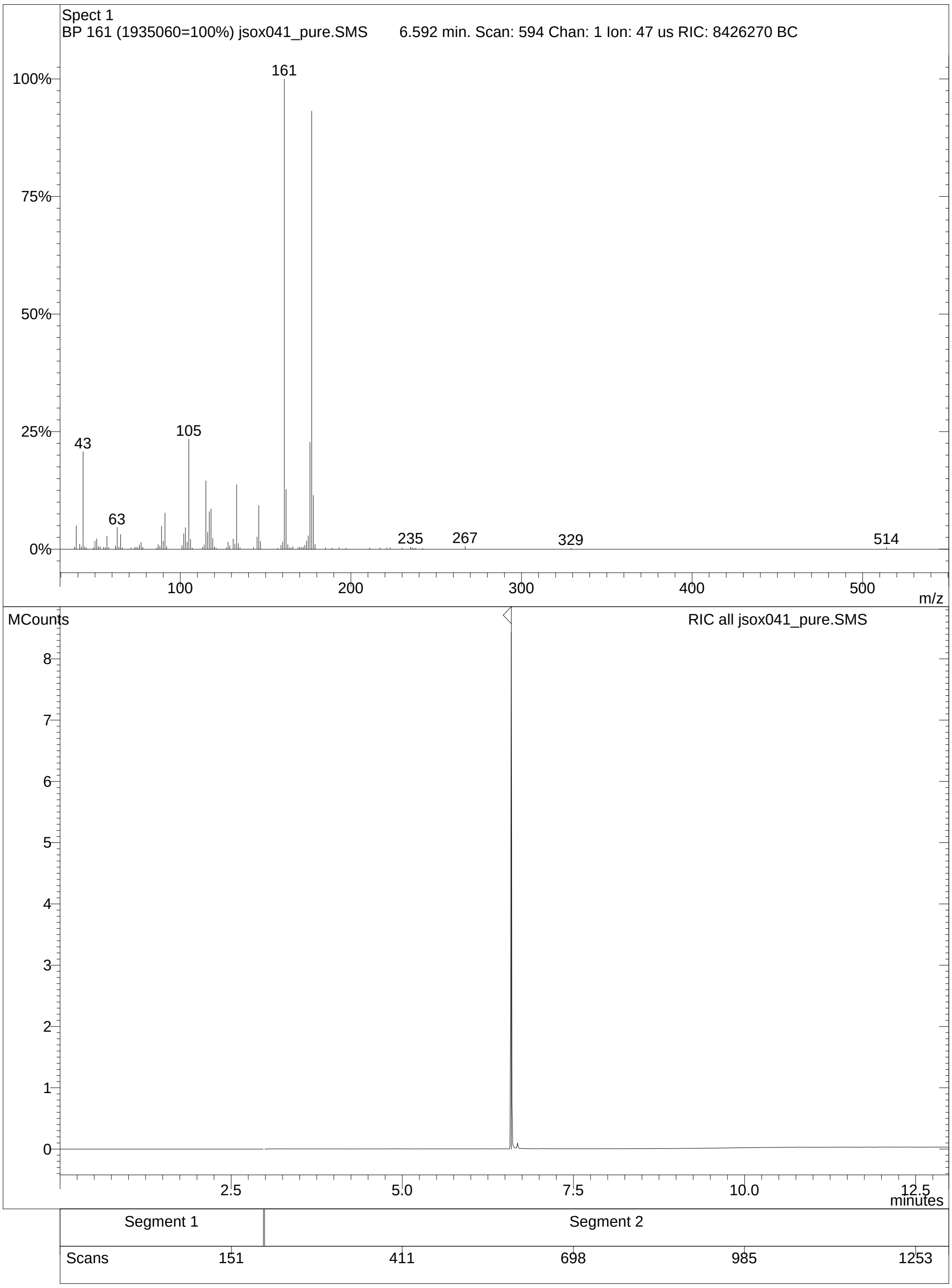

1-(p-tolyl)ethanone (4c)

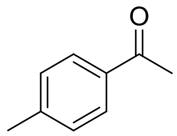

Chromatogram Plot

File: g:\gc-ms\jsox008_.sms
Sample: jsox008_f9 Operator: Org Farm Kemi
Scan Range: 1 - 1331 Time Range: 0.00 - 12.99 min. Date: 09/08/2008 18:02
Sample Notes: Routine

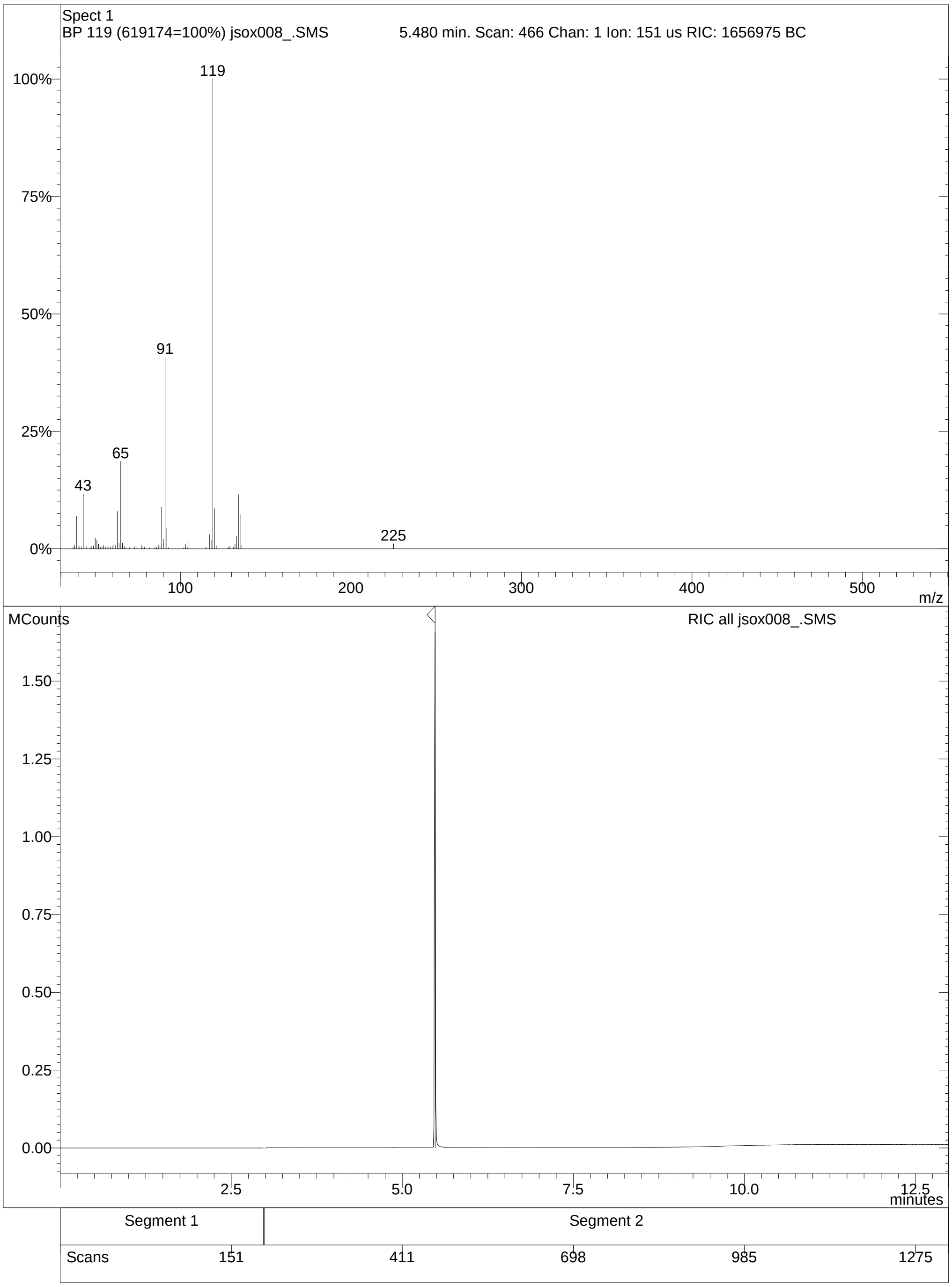

1-(4-bromophenyl)ethanone (4d)

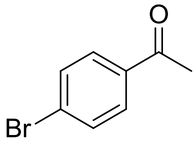

Chromatogram Plot

File: g:\gc-ms\jsox5060_.sms
Sample: JSOx5060_ Operator: Org Farm Kemi
Scan Range: 1 - 1159 Time Range: 0.00 - 11.16 min. Date: 10/26/2011 10:56
Sample Notes: Routine

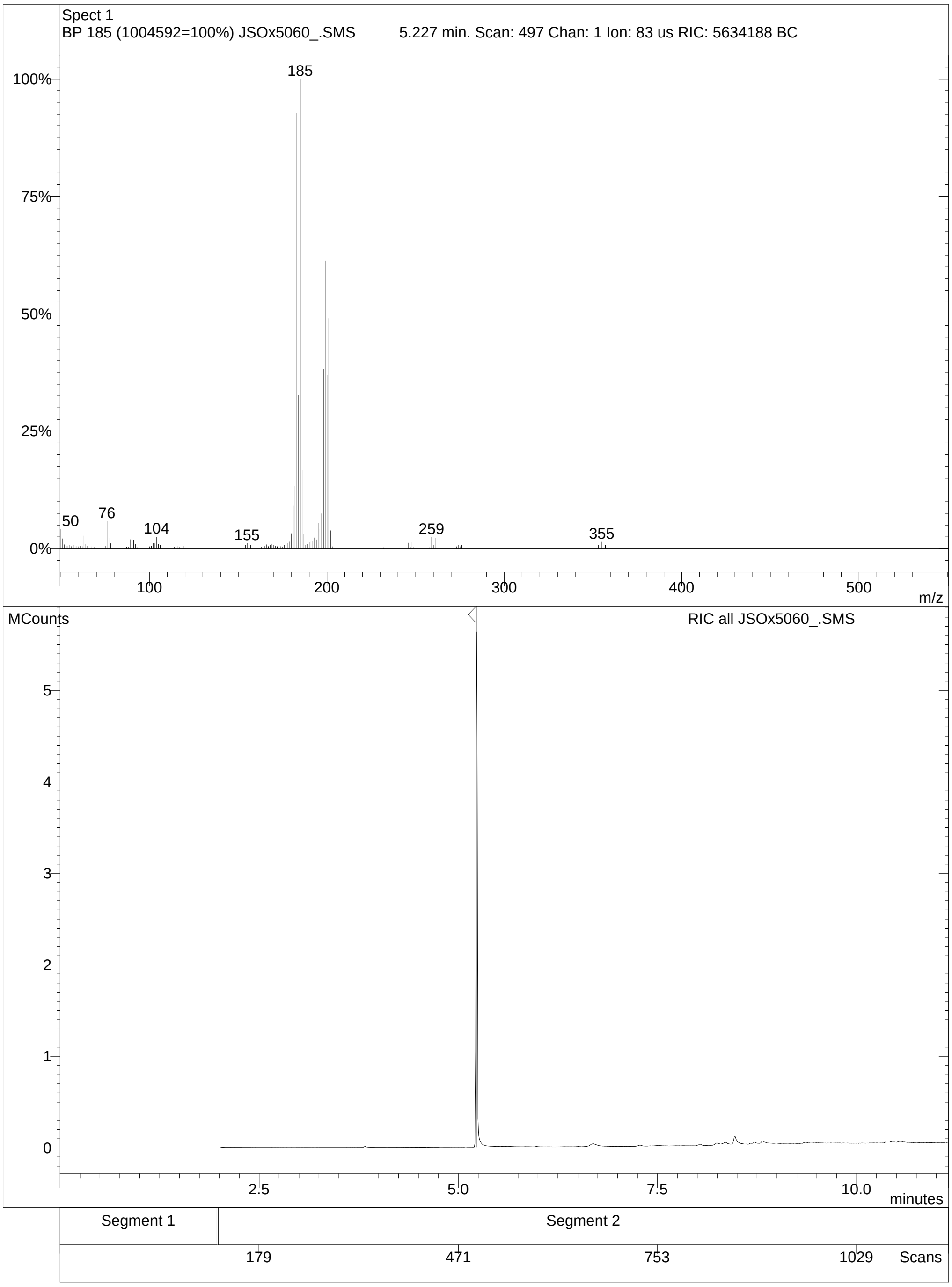

1, 4-diacetylbenzene (4e)

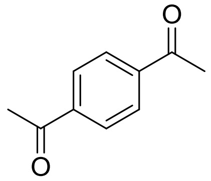

Chromatogram Plot

File: g:\gc-ms\jsox106_p.sms
Sample: JSOx106_p Operator: Org Farm Kemi
Scan Range: 1 - 1148 Time Range: 0.00 - 11.16 min. Date: 10/05/2010 14:44
Sample Notes: Routine

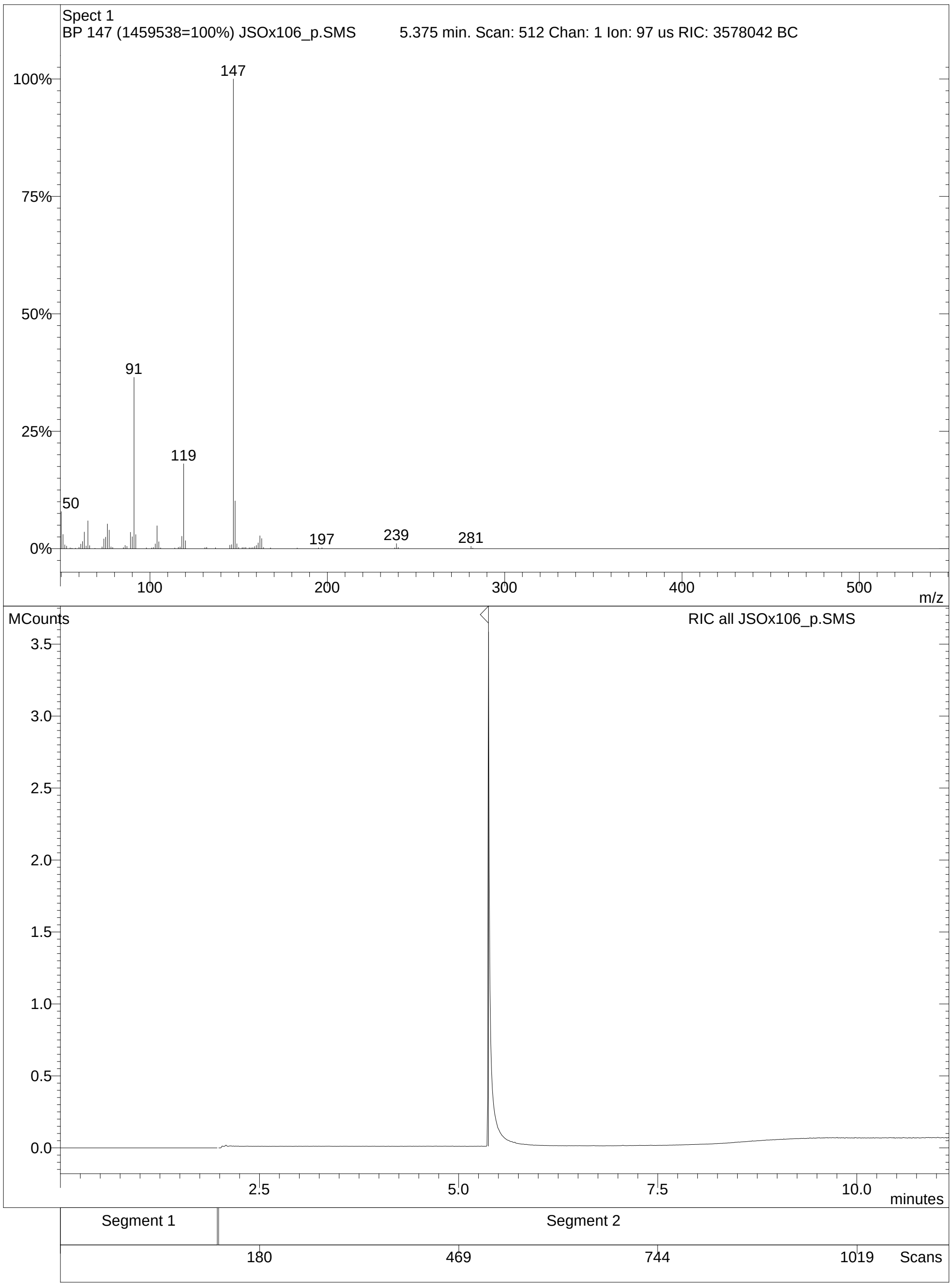

1-(4-(1-butoxyvinyl)phenyl)ethanone (3a)

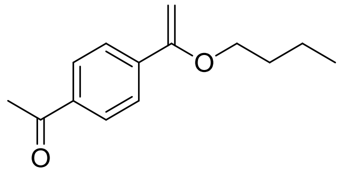

Chromatogram Plot

File: g:\gc-ms\jsox040_.sms
Sample: jsox040_f1 Operator: Org Farm Kemi
Scan Range: 1 - 1300 Time Range: 0.00 - 12.98 min. Date: 01/30/2009 16:11
Sample Notes: Routine

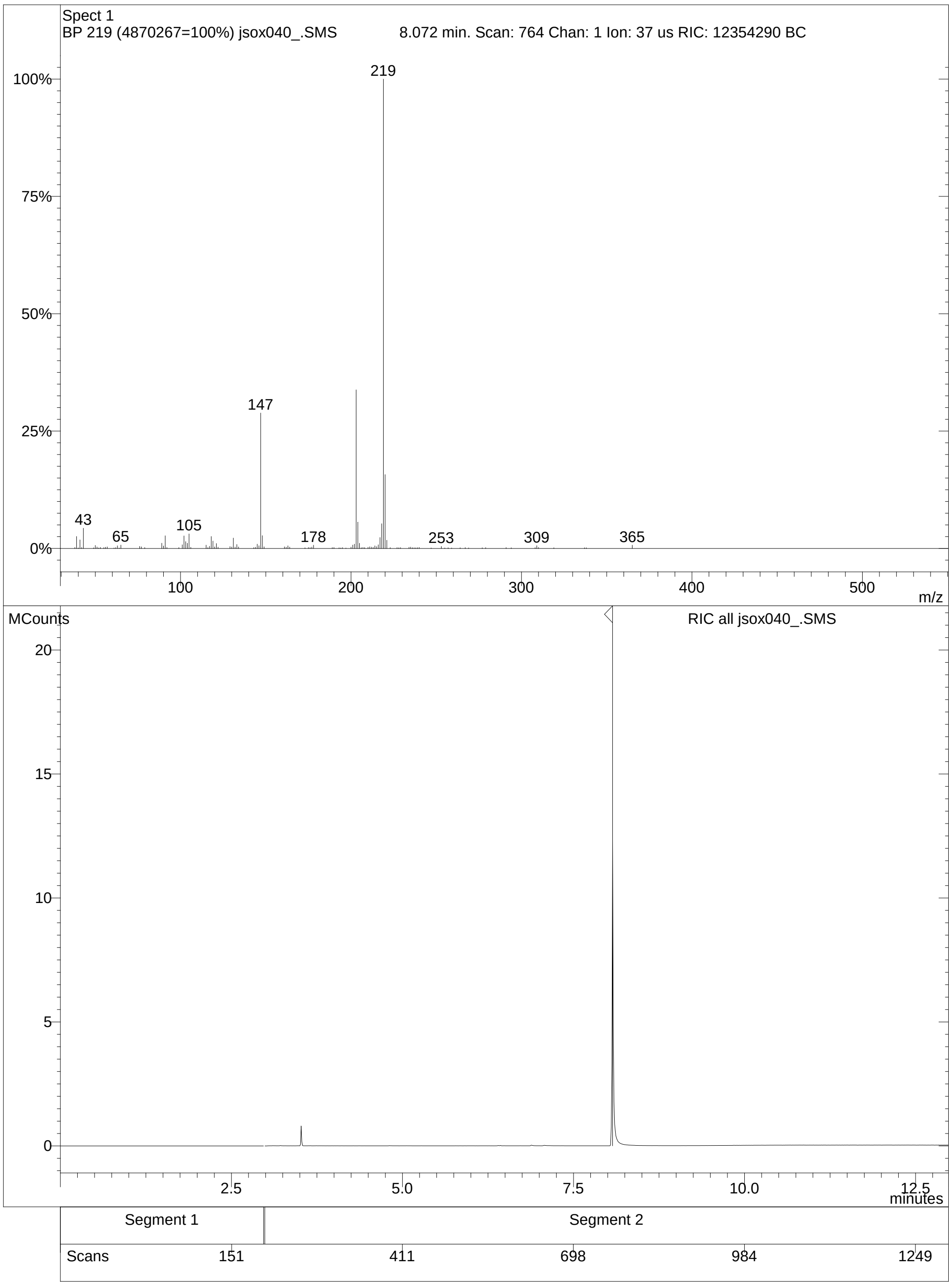

1-(4-(trifluoromethyl)phenyl)ethanone (4f)

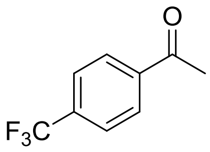

Chromatogram Plot

File: g:\gc-ms\jsox036_pure.sms
Sample: jsox036_pure
Scan Range: 1 - 1304 Time Range: 0.00 - 12.98 min.
Sample Notes: Routine

Operator: Org Farm Kemi
Date: 01/29/2009 17:24

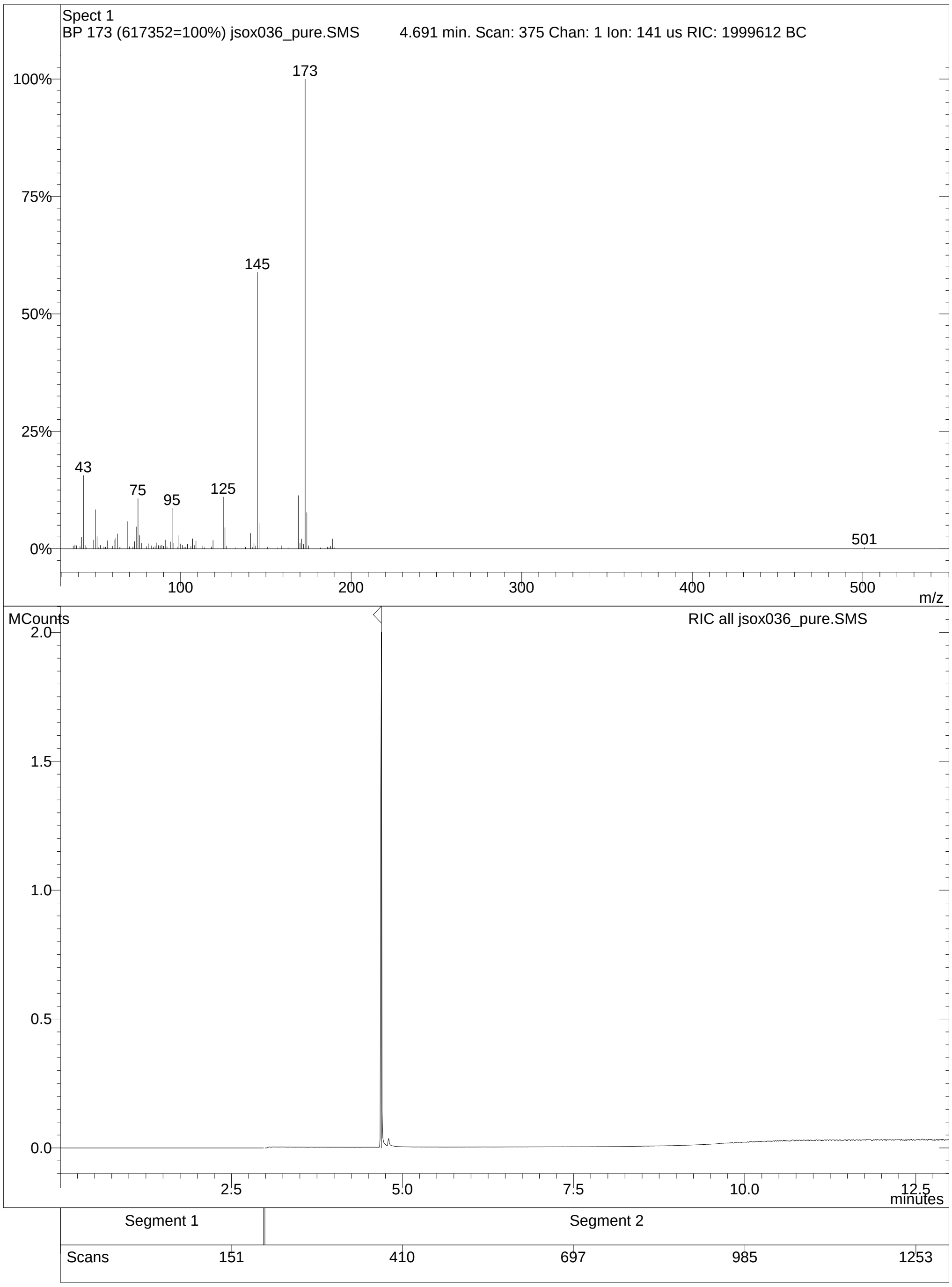

ESI-MS-(+) spectra

Typical ESI-MS spectrum using n-butyl acrylate in an reaction after 30 min at 40 °C.

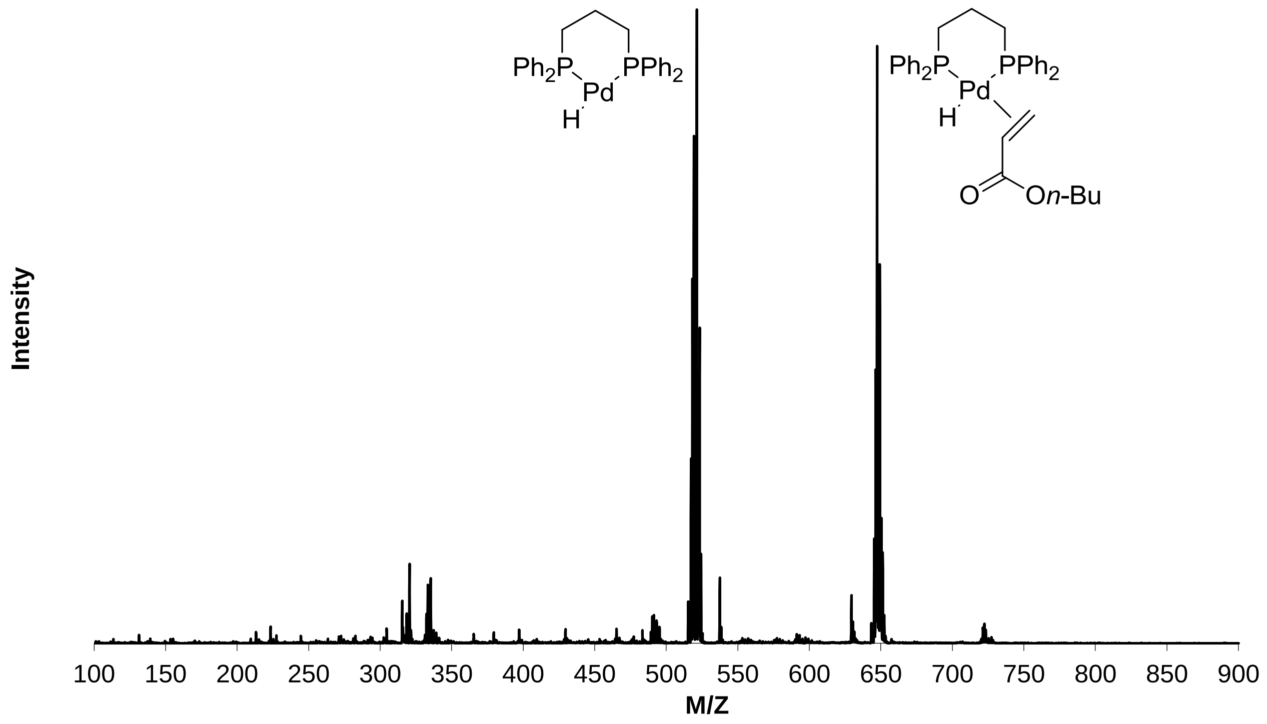

Supplement: Supplementary file 1 [file open0001-0140-SD1.pdf]
